# Exploring avocado consumption and health: a scoping review and evidence map

**DOI:** 10.3389/fnut.2025.1488907

**Published:** 2025-02-10

**Authors:** Stephen A. Fleming, Tristen L. Paul, Rachel A. F. Fleming, Alison K. Ventura, Megan A. McCrory, Corrie M. Whisner, Paul A. Spagnuolo, Louise Dye, Jana Kraft, Nikki A. Ford

**Affiliations:** ^1^Traverse Science, Mundelein, IL, United States; ^2^Department of Kinesiology and Public Health, Bailey College of Science and Mathematics, California Polytechnic State University, San Luis Obispo, CA, United States; ^3^Department of Health Sciences, Sargent College of Health and Rehabilitation Sciences, Boston University, Boston, MA, United States; ^4^College of Health Solutions, Arizona State University, Phoenix, AZ, United States; ^5^Department of Food Science, University of Guelph, Guelph, ON, Canada; ^6^Institute for Sustainable Food and School of Psychology, University of Sheffield, Sheffield, United Kingdom; ^7^Department of Animal and Veterinary Sciences, The University of Vermont, Burlington, VT, United States; ^8^Department of Medicine, Division of Endocrinology, Metabolism and Diabetes, The University of Vermont, Colchester, VT, United States; ^9^Avocado Nutrition Center, Mission Viejo, CA, United States

**Keywords:** *Persea americana*, public health, obesity, cardiovascular health, dietary patterns

## Abstract

**Objective:**

This scoping review evaluates the breadth of research on avocado intake and health, considering all populations and health outcomes (registered on Open Science Foundation at https://osf.io/nq5hk).

**Design:**

Any human intervention or observational study where effects could be isolated to consumption of avocado were included. A systematic literature search through April 2024 was conducted (PubMed, Web of Science, Scopus, and CENTRAL) and supplemented by backwards citation screening. Dual screening, data extraction, and conflict resolution were performed by three reviewers and an interactive evidence map was created.

**Results:**

After deduplication, 8,823 unique records were retrieved; 58 articles met inclusion criteria, comprising 45 unique studies (28 interventions, 17 observational studies). Studies were largely conducted in the United States or Latin America and generally included adults, with overweight/obesity, frequently with elevated lipid concentrations. Interventions assessed the impact of diets enriched in monounsaturated fatty acids, diets higher/lower in carbohydrates, or in free-feeding conditions. Larger amounts of avocados were used in interventions than commonly consumed in observational studies (60–300 vs. 0–10 g/d, respectively). Blood lipids, nutrient bioavailability, cardiovascular risk, glycemia, and anthropometric variables were the most common outcomes reported across all studies.

**Conclusion:**

Future recommendations for novel research include the study of: European, Asian, adolescent or younger, and senior populations; dose–response designs and longer length interventions; dietary compensation; and the need for greater replication. The results have been made public and freely available, and a visual, interactive map was created to aid in science translation. This evidence map should enable future meta-analyses, enhance communication and transparency in avocado research, and serve as a resource for policy guidance.

## Introduction

Consuming nutrient-dense foods is a key component of having a high-quality diet and maintaining general health and wellbeing throughout the lifespan. The 2020–2025 Dietary Guidelines for Americans recommend half of each meal comprise fruits and vegetables (1). The avocado is a nutrient-rich food whose intake is associated with improved diet quality (2, 3). One-third of a medium-sized avocado (50 g) contains approximately 80 kcals, 3.4 g fiber (11% daily value [DV]), 44.5 μg folate (10% DV), 0.73 mg pantothenic acid (15% DV), 85 μg copper (10% DV), 10.5 μg vitamin K (10% DV), 254 mg potassium (7.5% DV), and 4 mg of sodium (0.2% DV) (4, 5). Additionally, avocados have a fatty acid composition rich in monounsaturated fatty acids (MUFA) (6, 7). Recent research suggests that avocado intake may beneficially impact cognitive functions (8, 9) and skin health (10).

A meta-analysis estimated that every 1% energy replacement of carbohydrates with MUFA (in subjects without disturbances of lipid metabolism or diabetes) can reduce LDL cholesterol by 0.8 mg/dL, triacylglycerol by 1.7 mg/dL, and increase HDL by 0.7 mg/dL (11). To our knowledge, four meta-analyses have assessed the effect of avocados on total cholesterol, triacylglycerols, HDL and LDL cholesterol (12–15). Results between these meta-analyses were heterogenous, as 3/4 found reductions in total cholesterol (13–15), 1/4 found reductions in triacylglycerols (15), 2/4 found increases in HDL (12, 14), 3/4 found reductions in LDL cholesterol (13–15), and the remainder in each case demonstrating a neutral effect of avocado on the blood lipid of interest. Two meta-analyses concluded that avocado intake has minimal impact on body weight or body composition (12, 16), and James-Martin et al. (13) concluded that studies were too heterogenous to meta-analyze outcomes including anthropometrics, glycemia, blood pressure, oxidative stress markers, and cardiovascular disease (CVD) risk.

Despite several meta-analyses assessing avocado intake and health, variability in their methods (inclusion criteria, sub-group analyses, use or lack of observational data, and exclusion of non-English literature) and results complicate definitive conclusions that avocado intake affects circulating lipids (or most other health outcomes). An additional constraint in some reviews (12, 13, 15) was the inability to identify a dose–response effect, given the inclusion of studies that did not specify avocado intake or studies in which the effect could be attributed to foods co-administered with avocado. Establishing a dose–response effect would significantly contribute to the ability to understand how avocado consumption impacts health.

Although several systematic reviews on the topic have been published, each varied substantially in inclusion criteria (12–18). To our knowledge, no review has been conducted systematically with minimal restrictions on study design, duration, population, outcomes, and language. Therefore, the aims of this scoping review were to systematically summarize studies on avocado intake in a single, comprehensive evidence map that can be used to (1) aid in the characterization of participant populations, study designs, dietary patterns, outcomes, and avocado intake reported in the literature, (2) visualize the strengths and gaps of the research on avocados and health, (3) identify and generate ideas for future research, (4) educate the broader nutrition science community on what is known about avocado nutrition, and (5) support future meta-analyses. To maintain relevancy with public policy, this review adapted methods used by the USDA Nutrition Evidence Systematic Review (NESR) methodology (19), with some modifications to include numerous study types, those published in non-English languages, and trials conducted in any country.

## Methods

This scoping review and evidence map was conducted in accordance with PRISMA-ScR (20) recommendations for scoping reviews and was registered with Open Science Foundation (OSF) at https://osf.io/nq5hk. Contrary to a systematic or narrative review, it was not designed to synthesize the results of each study, but to summarize the quantity and characteristics of the available evidence.

### Search strategy

This review aimed to find, assess, and synthesize all intervention and observational studies that quantified avocado intake in humans and associated health outcomes. A systematic literature search was conducted through PubMed, Web of Science, Scopus, and CENTRAL on October 17th, 2023, with no date restrictions, and again on April 29th, 2024, date restricted to the time between searches. Searches included terms related to the intake of avocado and MUFA and are provided in full detail in the Supplementary methods. The electronic search was supplemented with backward citation screening of all in-scope articles. Backward citation screening was performed using Scite (Henderson, NV, USA)^1^ or Spidercite (Systematic Review Accelerator, Bond University, Gold Coast, Australia)^2^ to export all reference lists of in-scope articles, where possible. Exported reference lists were subsequently uploaded to Covidence (Melbourne, Australia)^3^ and underwent single-review screening (title/abstract and full-text). Where full reference lists could not be exported and imported into Covidence, reference lists were manually screened for inclusion.

### Eligibility criteria

The 2025 USDA NESR protocol for dietary patterns and growth, body composition, and risk of obesity (21) was modified to suit an evidence map approach and greater inclusivity of diverse populations (Table 1). Modifications related to an evidence map approach (and not a systematic review) included expansion of criteria by: including cross-sectional and case–control studies and excluding studies on allergenicity and methods validation; removing restrictions on publication dates, population health status, study duration, language, and country; expanding outcomes of interest; and requiring exposure to avocado pulp (not an extract or oil thereof) to be quantified, statistically analyzed with relation to a health outcome, and isolatable in multi-component interventions. Modifications related to inclusivity were inclusion of studies on populations of any health status and those published in any language or country regardless of their Human Development Index, given numerous studies have been published in countries where English is not the primary language (especially Mexico).

**Table 1:** Eligibility criteria.

NESR protocol criteria^1^	Amendments to NESR protocol
**Study design** Include: randomized controlled trials (RCTs, such as individual, cluster, and crossover trials), non-RCTs, observational cohort studies (e.g., prospective or retrospective), nested case–control studies, and mendelian randomization studies. Exclude: uncontrolled trials, cross-sectional studies, ecological studies, case–control studies, reviews, and modeling and simulation studies.	Include: cross-sectional and case–control studies Exclude: studies on allergenicity or methods validation.
**Publication date** January 1980–May 2023	No date restrictions
**Population study participants and life stage** Children and adolescents (≥ 2 and < 19 years) Adults and older adults (> 19 years) Individuals during pregnancy/postpartum	Humans of any age
**Population health status** Studies that exclusively enrolled participants not diagnosed with a disease OR studies that enrolled some participants: diagnosed with a disease; diagnosed with a disorder that affects feeding/eating or growth (e.g., autism spectrum disorder, attention-deficit/hyperactivity disorder, eating disorder); with severe undernutrition, failure to thrive/underweight, stunting, or wasting; who became pregnant using Assisted Reproductive Technologies; with multiple gestation pregnancies; receiving pharmacotherapy to treat obesity; pre- or post-bariatric surgery; and/or hospitalized for an illness, injury, or surgery	No restrictions
**Intervention/Exposure** Consumption of avocado. Multi-component intervention in which the isolated effect of the intervention of interest on the outcome(s) of interest is provided or can be determined despite multiple components	Studies must meet all of the following criteria: Avocado intake is quantified in a form convertible to g/d (e.g., % energy, % of fat, servings, frequency, etc.) Consumption of avocado as a food, and not a supplement/extract. Avocado is the only food whose intake was manipulated (aside from the foods/nutrients that avocado displaced). Exclude: Components of avocado such as avocado oil, leaves, peel, seed, extracts thereof, and powdered forms.
**Comparator** Consumption of different amounts of avocado	Consumption of different amounts of avocado, or an alternative source of dietary fat or macronutrients.
**Outcome(s)** NA	Include: Any outcomes related to human health and statistically analyzed for their relationship to avocado intake. Exclude: Effects of behavioral/dietary patterns on avocado intake.
**Study duration** Intervention length ≥ 12 weeks (in children, adolescents, adults, and older adults only)	No restrictions
**Sample size** No restrictions	No amendments
**Publication status** Peer-reviewed articles published in research journals	No amendments
**Language** Published in English	No restrictions
**Country** Studies conducted in countries classified as high or very high on the Human Development Index the year(s) the intervention/exposure data were collected	No restrictions.

^1^These criteria represent an adaptation of the 2025 NESR protocol for Dietary Patterns and Growth, Body Composition, and Risk of Obesity: A Systematic Review Protocol (21) and USDA NESR Methodology manual (19).

### Study screening, selection, and data extraction

Dual screening at the title/abstract and full-text levels was conducted by three reviewers in Covidence^4^. Discrepancies were resolved by discussion between reviewers or by a senior reviewer. Two reviewers independently extracted all data from each study using pre-defined sheets in Microsoft Excel, and conflicts were resolved through discussion or by a senior reviewer. No automation or AI-based tools were used for classification/categorization during screening or data extraction. Variables were extracted at either the per-study level (e.g., bibliographic information, study design, sample size, country of origin, funding source) or on a per-group level (subject characteristics, avocado and nutrient intakes, and all health outcomes measured). A complete list of all variables extracted is available in Supplementary Tables S1, S2. Data were extracted from both the main text, tables, figures, and all relevant Supplementary material. Only values (e.g., means and variation) related to avocado intake, BMI, age, sex, and blood lipids were extracted, to enable characterizing the participants studied. Where possible, data were extracted as described by the authors and re-classified or transformed for data analysis and visualization.

### Data analysis and visualization

Data cleaning, transformation, descriptive analysis, and visualization were performed manually in Excel and Tableau Cloud (Tableau Server Version 2024.1.0). To avoid double-counting secondary analyses of the same study, each publication was classified according to whether it represented the “primary” report and if subsequent publications were conducted on unique populations. References to the count of primary reports indicate whether a given variable was assessed in the first or any subsequent publication from the same primary report unless specified otherwise. For example, the clinical trial NCT01235832 published by Wang et al., in 2015 and 2020 (22, 23) reported the same population but different outcomes. This clinical trial was treated as a single “primary report” rather than two. Conversely, the *Persea americana* for Total Health Study (NCT02740439) published initially by Edwards et al., 2020 (8) is detailed in three other publications (24–26), each of which differed slightly in the subject populations analyzed. Each publication’s sample size, subject characteristics, and outcomes were treated independently, whereas variables common to all four publications (e.g., study design, country of study) were treated as a single report. For simplicity, nearly all data reported in this manuscript were treated as a single report when multiple publications from the same data source (e.g., a survey, prospective cohort, clinical trial) exist, unless specified otherwise.

Similarly, multi-categorical information, such as a study on participants in both the United States (U.S.) and Canada (27) was stored in a single cell (e.g., U.S.; Canada) and then split into multiple rows. Thus, the percentage of primary reports that describe a given multi-categorical variable (e.g., funding sources, weight status, age of participants) may add to over 100%, as the categories are not mutually exclusive. Group and sub-group information (e.g., avocado intake in treatments A and B in males and females) were stored on multiple rows. All counts presented here were de-duplicated but not in the raw database. Unit conversions for avocado intake (e.g., from % energy, frequency, or other form into g/d) are listed within the database itself and in Supplementary Tables S3, S4. Protocols, Supplementary material, interactive visualizations, and raw data are freely available at https://github.com/Traverse-Science/Avocado-Evidence-Map, and archived at https://doi.org/10.5281/zenodo.12824839.

## Results

**Figure 1:**
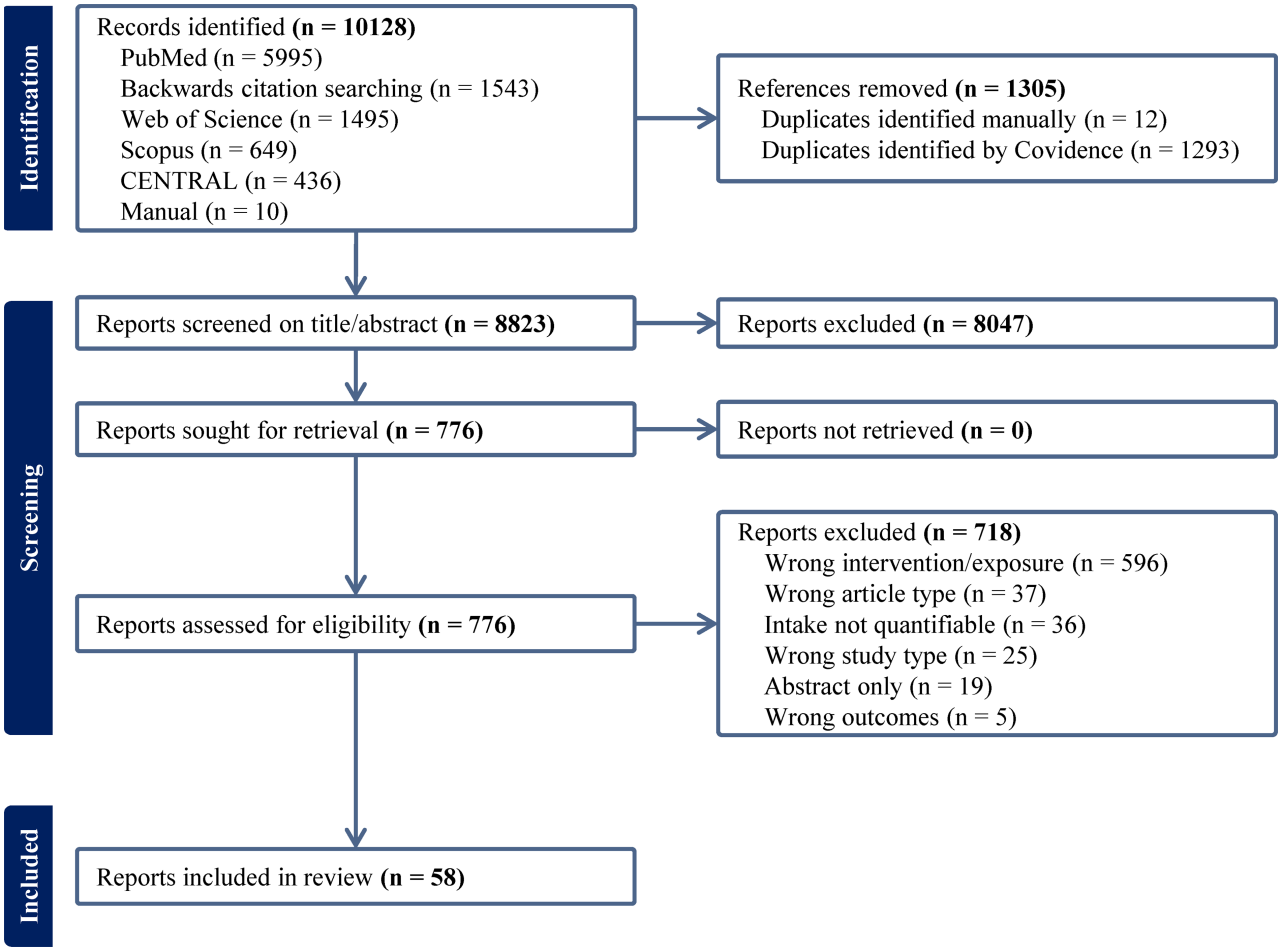
PRISMA diagram for searches performed on 10/17/2023 and 04/29/2024 (mm/dd/yyyy).

From all sources, 8,823 unique references were identified. At the title/abstract level, 8,047 references were excluded, leaving 776 articles for full-text screening. After the exclusion of 718 articles, 58 articles met the inclusion criteria (Figure 1). Most exclusions (596/718, 83%) were made because studies examined an intervention or exposure irrelevant to this review. Thirty-six studies included the study of avocado, but intake was either not specified at all or in insufficient detail. After adjusting for multiple articles from the same trial, 58 publications comprised 53 analyses of unique populations from 45 primary reports (28 interventions, 17 observational studies). Notably, there were numerous studies on the topic of avocado that were excluded from this review. Excluded studies described in previous reviews (12, 13, 15, 18, 28, 29) were excluded based on (1) intake not being quantified (30, 31), (2) provision of avocado with olive oil (32, 33) or olive oil and almonds (30, 31), and (3) single-arm trial designs (34–36). We additionally found other ineligible articles that described the provision of avocado with kiwi (37), nuts (38), multiple other foods (39, 40), lack of a control group (41), or as a pill-based supplement and not part of the diet (42). Although excluding these reduced the size of the evidence map by over 10 studies, it focused the review of interventions to those studies whose reported effects could be isolated to experimental manipulation of avocado intake alone. A list of excluded studies are available in Supplementary Data Sheet 1.

Among observational trials, there were five prospective cohorts (Multiethnic Cohort Study, Multi-Ethnic Study of Atherosclerosis, Nurses’ Health Study I and II, and Health Professionals Follow-up Study) that were analyzed in multiple articles (3, 43–51) (Table 2). Various sampling dates of the National Health and Nutrition Examination Survey were reported, but these were considered unique given each article (3, 50, 51) investigated different subject populations. Six clinical trials were reported across multiple publications, but these differed in whether they represented a secondary analysis of the same population (23, 52–54) or an analysis of a unique population (24–26, 55, 56) (Table 2).

**Table 2:** Publications related to the same primary report.

Primary report	Additional publications	Unique population^1^
Observational studies		
Multiethnic Cohort Study (MEC)	Monroe et al., 2007 (43) Cheng et al., 2024 (80)	Yes
Multi-Ethnic Study of Atherosclerosis (MESA)	Wood et al., 2023 (45)	Yes
	Cheng et al., 2023 (46) Carris et al., 2024 (79)	Yes
Nurses’ Health Study (NHS), NHS II, and Health Professionals Follow-up Study (HPFS)	Pacheco et al., 2022a (47) NHS (1986–2016), HPFS (1986–2016)	Yes
	Ericsson et al., 2022 (48) NHS (1986–2014), HPFS (1986–2016)	Yes
	Borgi et al., 2016 (49) NHS (1986–2014), NHS II (1999–2011) HPFS (1986–2010)	Yes
National Health and Nutrition Examination Survey (NHANES)	Fulgoni et al., 2013 (50) 2001–2008	Yes
	O’Neil et al., 2017 (3) 2001–2012	Yes
	Cheng et al., 2021 (51) 2011–2014	Yes
Interventions		
Effects of Avocado Intake on the Nutritional Status of Families Trial (NCT02903433) Pacheco et al., 2021 (71)	Pacheco et al., 2022b (55) VanEvery et al., 2023 (56)	Yes
	Allen et al., 2023 (52)	No
NCT01235832 Wang et al., 2015 (22)	Wang et al., 2020 (23)	No
NCT01271829 Wien et al., 2013 (65)	Haddad et al., 2018 (53)	No
NCT02479048 Park et al., 2018 (86)	Zhu et al., 2019 (54)	No
*Persea Americana* for Total Health Study (NCT02740439) Edwards, 2020 (8)	Hannon et al., 2020 (24) Khan et al., 2021 (25) Thompson et al., 2021 (26)	Yes
Habitual Diet and Avocado Trial (NCT03528031) Lichtenstein et al., 2022 (72)	Petersen et al., 2024 (2)	No

^1^An article was considered distinct (i.e., unique) from the primary report if it analyzed a different population, indicated by an analytical sample size different from the primary report, analysis on sub-groups, analysis on a subset of the population from the primary report, or sampled populations on a different time-scale. These may or may not have analyzed the same outcomes as the primary report.

### Publishing trends

**Figure 2:**
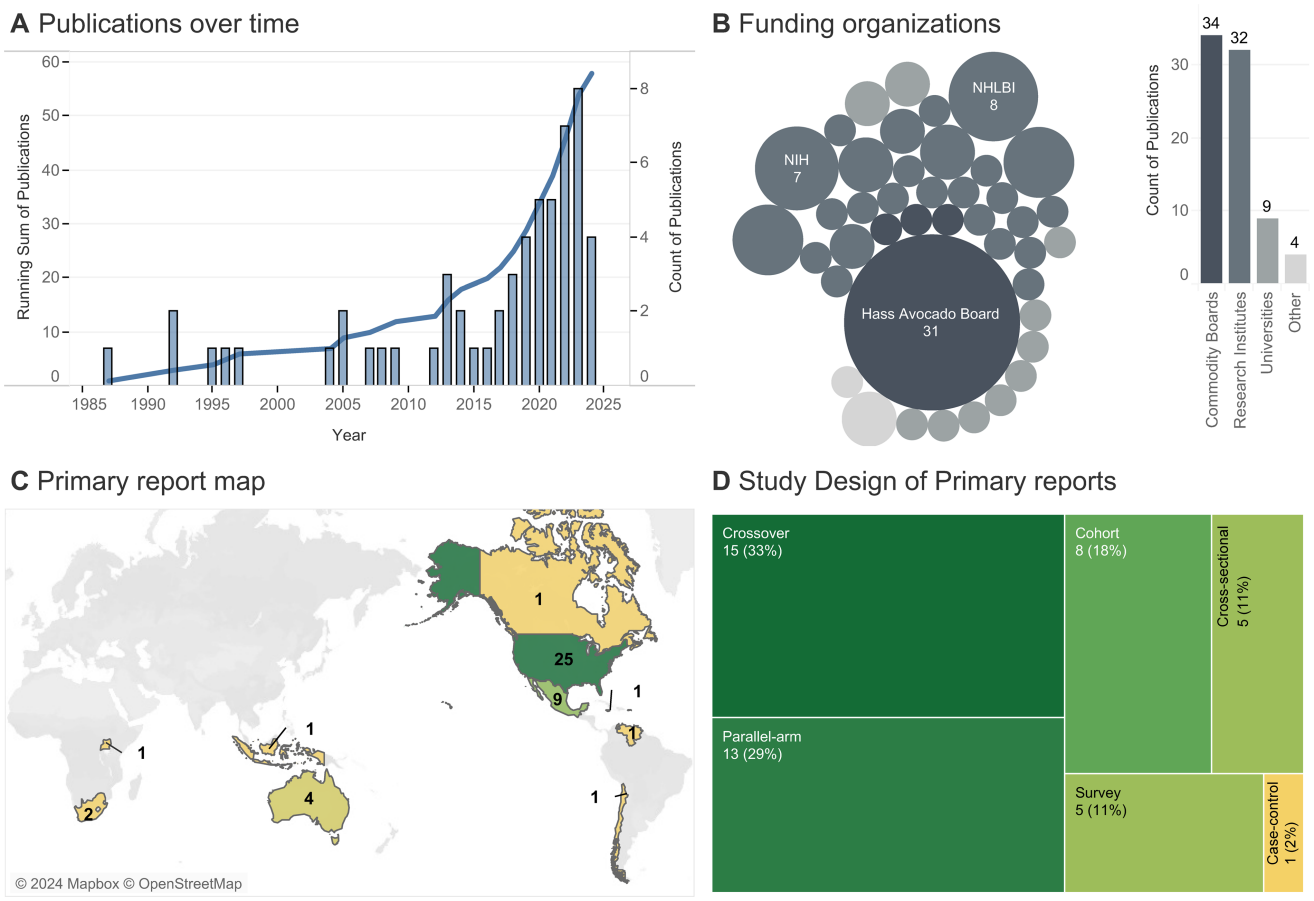
Publishing trends. **(A)** The line indicates the cumulative number of publications per year (totaling 58), with bars detailing yearly publications. **(B)** Each bubble represents a unique funding source (one article could come from multiple sources), with the size indicating the number of articles associated with a given funding source (smallest bubble = 1 article). **(C)** Number of primary reports (totaling 45) conducted on participants from a given country (one primary report was conducted in both the U.S. and Canada); color indicates the number of studies. **(D)** Number of primary reports of a given study design (one primary report analyzed as both a cross-sectional and a cohort).

The earliest in-scope research on avocados began in 1985 (Figure 2), though, to our knowledge, the first study published on avocado intake and health was by Grant et al., 1960 (34); however, that study was out-of-scope given it lacked a control group. A steep rise in publications per year began in 2017, with 29/58 (50%) articles published between 2019–2023. The top five most commonly reported funding organizations (studies could have multiple funding sources), in order, were the Hass Avocado Board (which includes the Avocado Nutrition Center), the National Heart, Lung, and Blood Institute (NHLBI), NIH, National Institute of Diabetes and Digestive and Kidney Diseases (NIDDK), and the USDA. In total, 58% of articles reported funding from commodity boards, 55% from research institutes, 16% from universities, and 7% from other sources (e.g., hospitals or companies). The majority of primary reports were conducted on participants in the Americas (U.S., Mexico, Canada, Jamaica, Chile, and Venezuela) (Figure 2). No studies were conducted in Europe and only one was conducted in Asia (in Indonesia). A small number of studies were conducted in South Africa, Uganda, and Australia. The geographical trend matches the funding source, as there were no funding sources from European/Asian commodity boards, and the only research institute in Asia (Korean International Cooperation Agency) funded a study in South Africa (57). The majority of primary reports were interventions (28/45, 62%) of crossover or parallel-arm design, with the remainder observational trials, typically a prospective cohort (8/45, 18%) or cross-sectional/survey study (10/45, 22%).

### Participant characteristics

**Figure 3:**
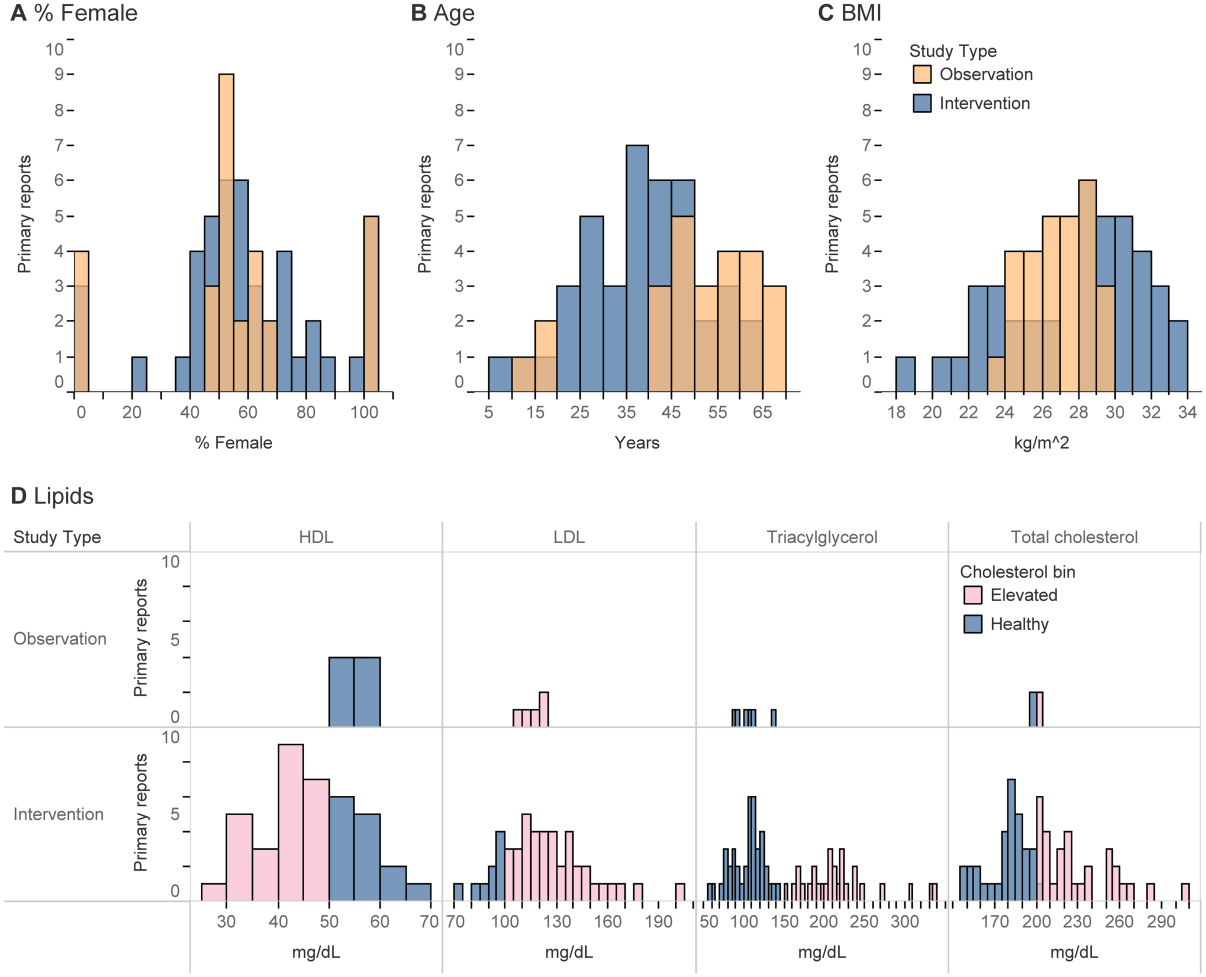
**(A-D)** Histograms represent the number of primary reports that described a given participant characteristic (using an absolute value, mean, or median) of any analytical group/sub-group at any point during a study. **(D)** For lipids, values were classified as healthy if in the ranges of 125–200 for total cholesterol, < 100 for LDL, > 50 for HDL, and < 150 for triacylglycerols (all in mg/dL).

The intended population was usually described as “healthy” (no diagnosis of a medical disease/disorder) or described in vague terms such that it was assumed to be about the general population (Table 3). Only seven studies were intentionally designed to study participants with elevated blood lipids or those diagnosed with or at risk for type 2 diabetes (T2D). Regardless of the intent, the majority of studies were conducted in adults, fairly balanced in sex with a skew toward females, predominantly in those with overweight/obesity, and those with elevated blood cholesterol (Table 3; Figure 3). Interventions most frequently included individuals of middle (~35–50 yrs) or college ages (~20–35 yrs), and observational studies primarily described participants ages 40 yrs. or older (Figure 3). Intervention studies exhibited a bi-modal-like distribution with participant BMI clustered in ranges of 23–26 or 28+ kg/m^2^, whereas observational studies reported ranges between these two peaks, from 24–29 kg/m^2^ (Figure 3).

**Table 3:** Participant characteristics.

Characteristic	Count of primary reports n (% total, column-wise)	
	Interventions 28 (100)	Observations 17 (100)
Intended population^1^		
Healthy/general population	18 (64)	15 (88)
Elevated cholesterol/triacylglycerols^2^	6 (21)	0
T2D or insulin resistance	2 (7)	1 (6)
Other^3^	3 (11)	1 (6)
Age, yrs^4^		
Adolescent or younger (< 18)	2 (7)	1 (6)
Adult (≥ 18, < 65)	25 (89)	13 (76)
Senior (≥ 65)	0	3 (18)
Sex^4^		
Mostly female (> 60% female)	14 (50)	10 (59)
Balanced (40–60% female)	14 (50)	10 (59)
Mostly male (< 40% female)	5 (18)	4 (24)
BMI,^4,5^ kg/m^2^		
Underweight (< 18.5)	0	0
Healthy weight (≥ 18.5, < 25)	7 (25)	4 (24)
Overweight or obese (≥ 25)	17 (61)	13 (76)
Blood lipid concentrations^1,2,4–6^		
Healthy (by at least one metric)	17 (61)	4 (24)
HDL cholesterol	9 (32)	4 (24)
LDL cholesterol	4 (14)	0
Total cholesterol	14 (50)	2 (12)
Triacylglycerols	13 (46)	3 (18)
Elevated (by at least one metric)	17 (61)	3 (18)
HDL cholesterol	13 (46)	0
LDL cholesterol	15 (54)	3 (18)
Total cholesterol	12 (43)	2 (12)
Triacylglycerols	9 (32)	

T2D, type 2 diabetes. ^1^Intended population was categorized according to subject health status at baseline/time of analysis, not at follow-up. If participants were not explicitly stated as healthy, the “general population” was assumed to be the target, unless inclusion criteria specified otherwise or authors reported intentional recruitment of participants with a specific disease/disorder. ^2^Given the intended population was not always specified, some trials did not consider their participants to have elevated blood lipids. This is shown by the discrepancy between the five primary reports (22, 23, 58–61) that explicitly recruited participants with dyslipidemia compared to 21 primary reports that reported at least one group of participants with elevated blood lipids at some point during the study. ^3^“Other” category includes conditions such as hookworm (57), metabolic syndrome (67, 77), and prostate cancer (82). ^4^Categories are not mutually exclusive, and not all studies reported each variable. Where values do not sum to 100% was typically in relation to incomplete reporting, or reporting of the same variable over multiple sub-groups (e.g., BMI in multiple age-groups, or lipids across males/females). ^5^Values represent the number of primary reports that described a given characteristic (using an absolute value, mean, or median) of any group/sub-group at any point during the study. For example, a hypothetical study wherein an analytical group had a high value (e.g., BMI, cholesterol) at baseline that lowered after follow-up would be counted in multiple ranges. Values were presented this way as not all studies reported participant characteristics at baseline. ^6^Classified according to any of HDL, LDL, or total cholesterol, where healthy was designated (all in mg/dL) as 125–200 total cholesterol, < 100 LDL cholesterol, > 50 HDL cholesterol, and < 150 triacylglycerols, using ranges set at https://medlineplus.gov/cholesterollevelswhatyouneedtoknow.html. Although HDL > 40 is considered healthy for males, most studies did not separate the data by sex. Healthy concentrations were based on guidelines for women given greater representation of females across studies.

Despite the relatively small number of primary reports (22, 23, 58–61) that explicitly recruited participants for their elevated blood lipids (18%, Table 3), all interventions that reported blood lipid concentrations (17/28 primary reports) detailed elevated blood lipid concentrations by at least one metric (HDL, LDL, total cholesterol, or triacylglycerols) at some point during the study (Table 3). Participants were most likely to be rated as having elevated lipids, or conversely least likely to be rated as healthy, according to HDL and LDL concentrations. These characteristics were infrequently reported among observational studies.

### Design of dietary interventions

There were four different designs of dietary control among intervention trials: (1) Constant macronutrient intake between groups (on a % energy basis) with enrichment of MUFA from avocado, (2) Higher vs. lower carbohydrate/fat intake (on a % energy basis) with avocado as a primary fat source, (3) Free feeding of avocado with no control over macronutrient intake or energy consumption, and (4) Studies not concerned with macronutrient intake (Table 4). Habitual intake studies represent the most translatable designs as they allow for potential dietary compensation; however, it can be difficult to isolate whether the resulting changes can be attributed to the intake of avocado itself independent of dietary compensation.

**Table 4:** Design of macronutrient intake in interventions.

Count of primary reports of interventions, n (%) 28 (100)	Comparator	
**Experimental control of energy from macronutrients**	**Avocado consumption with replacement of other foods** 19 (68)	**Avocado consumption on top of habitual/other dietary intake** 12 (43)
Macronutrients constant between groups 8 (29)	**MUFA enrichment** 8 (29)	1^1^ (4)
Macronutrients allowed to deviate between groups 16 (57)	**Higher vs lower CHO** 9^2^ (32)	**Free feeding** 9^2^ (32)
Control of macronutrients not reported 5 (18)	3 (11)	2 (7)

CHO, carbohydrates. ^1^Macronutrients (on a % energy basis), but not energy, were held constant (53, 65), hence why it’s possible to maintain equivalent macronutrient intake between groups without replacement. This primary report also included a group that controlled intake with replacement of other foods and is represented under the “MUFA enrichment” group. ^2^Two studies included both an *ad libitum* habitual intake group and an arm with controlled intake with replacement of other foods and are represented in both comparator columns (6, 59).

**Figure 4:**
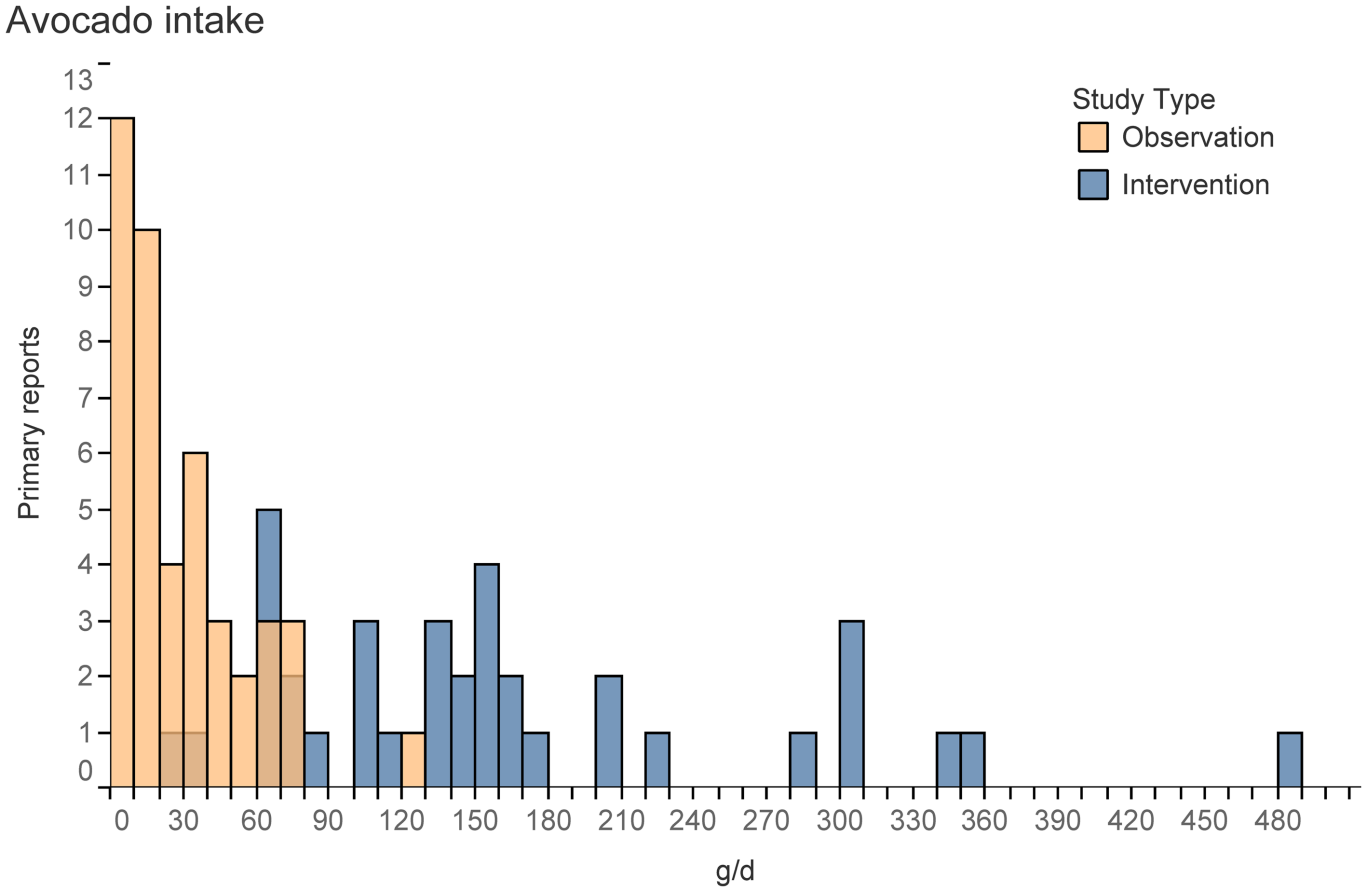
Histogram represents the number of primary reports that described a given level of avocado intake (e.g., using an absolute value, mean, median, or quantiles) of any group/sub-group at any point during a study. Excludes groups with non-zero intakes from control groups or non-consumers.

The most common comparators included the consumption of some avocado to no avocado (Table 5). However, observational trials more frequently included dose–response evaluations. Among the observational trials, most primary reports (44%) assessed the intake of whole, raw avocado, but not avocado from all food sources. In such cases, participants who consumed guacamole but not whole avocado would be considered “non-consumers” of avocado. Only 2/17 observational primary reports (62, 63) estimated intake from all food sources of avocado. This is likely why the most common intake of avocados was reported between 0–10 g/d (7% of an avocado/d, assuming 1 avocado = 150 g) among observational trials (Figure 4). Among interventions, intakes of avocado spanned between 30–490 g/d (or per meal, in the case of acute studies) but was most commonly 60–70, 150–160, 210–220, or 300–310 g/d (Figure 4). Such amounts are roughly equivalent to 0.5, 1, 1.5, and 2 whole avocados/d (assuming the average avocado weighs 150 g). The difference in exposure to avocado varies by upwards of 5-10x between observational and intervention trials.

**Table 5:** Dietary characteristics.

Characteristic	Count of primary reports, n (%)	
	Interventions	Observations
Comparator^1^	28 (100)	17 (100)
No avocado	26 (93)	8 (47)
Less avocado	1 (4)	3 (18)
Dose–response	1 (4)	8 (47)
Avocado Source^1^		
Whole, raw avocado only	NA	1 (6)
Avocado and guacamole	NA	5 (29)
All sources	NA	3 (18)
Not specified	NA	10 (59)
As directed (in designed meals or as desired)	28 (100)	NA
Intended energy balance of participants^1,2^		
Not applicable (acute studies)	8 (29)	NA
No intent (*ad libitum*, free-living, habitual)	9 (32)	NA
Weight loss	4 (14)	NA
Weight maintenance	7 (25)	NA
Weight gain	0	NA
Intended energy balance between groups^1,2^		
No intent	8 (29)	NA
Hypocaloric	0	NA
Isocaloric	18 (64)	NA
Hypercaloric	5 (18)	NA
Nutrient intake ascertainment method^3^		
FFQ or custom questionnaire	2 (7)	11 (65)
24-h dietary recalls	2 (7)	6 (35)
3-7d food/diet records	2 (7)	0
Prescribed/controlled	9 (32)	0
Not applicable	9 (32)	0
None reported	4 (14)	0

FFQ, food frequency questionnaire. ^1^This is a multi-categorical option, thus rows within this category may not sum to 100%. ^2^Energy balance of the participants and balance between groups were classified according to the described intent of the authors, and not the actual energy balance achieved in the study. Actual energy balance was frequently not possible to determine or may have differed from what was intended, especially in the case of free-feeding studies. ^3^Reporting of a method for ascertaining nutrient intake does not indicate whether nutrient intakes themselves were analyzed and/or reported.

Among the interventions, the intended energy balance of participants was either not relevant (29%, in acute studies), not explicitly controlled (32%, among habitual intake or *ad libitum* designs), or either a weight maintenance (25%) or weight loss (14%) design (Table 5). Sixty-four percent of interventions controlled intake such that comparisons between groups were isocaloric, 29% allowed energy intake to deviate (purposefully or due to dietary compensation), and a small proportion (18%) assessed avocado intake in a hypercaloric design. Such hypercaloric designs were more common in bioavailability or acute intake studies comparing avocado intake to the lack thereof or to different foods (53, 64–68).

Observational trials used either a food frequency questionnaire (FFQ) or food/diet record, and 88% of observational trials described the energy intake of participants compared to 65% of interventions. Intervention trials usually controlled nutrient/food intake through a prescribed diet, did not report nutrient intake at all (14%), or did so using a FFQ, 24-h recall, or 3-7d food record (7%) (Table 5).

**Figure 5:**
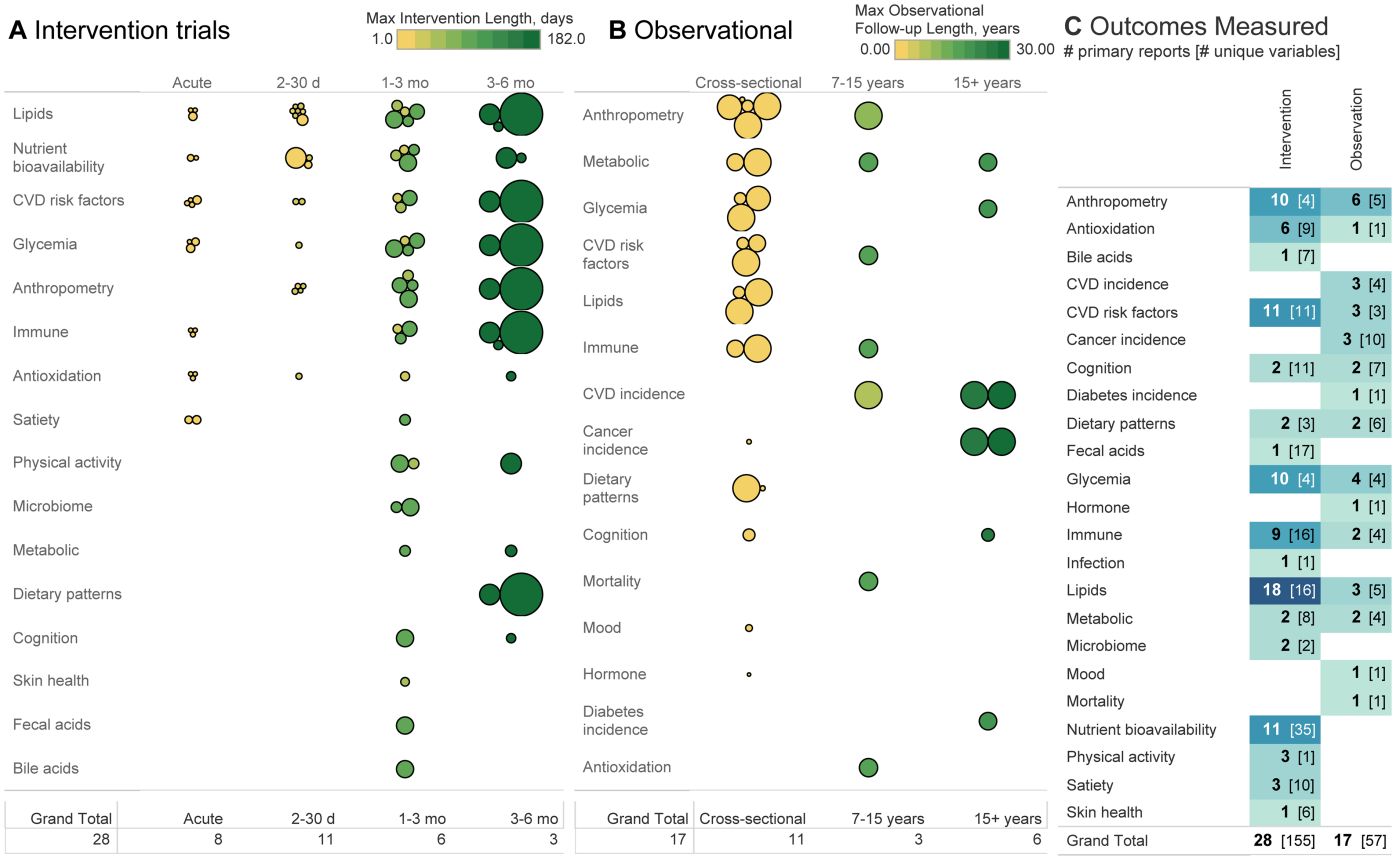
**(A, B)** Each bubble represents a primary report, organized according to sample size (size of bubble), length of intervention/follow-up (observational cohorts may have multiple follow-up periods according to different analyses and may thus be listed over multiple follow-up lengths), and a broad classification of the outcomes studied. **(C)** Heatmap depicts the number of primary reports and the number of distinct variables measured within the subdomain listed. For example, 10 interventions measured anthropometric outcomes using four different variables (e.g., weight, BMI, body fat, and body size). Each of those four variables may have then used a different method of measurement (not depicted), such as describing body fat using total fat % or mass of visceral adipose tissue. The greater the number in brackets, the greater the heterogeneity between studies in variables reported and methods of measurement used.

Reporting nutrient intake was usually completed (Table 6), with 75% or fewer primary reports of interventions reporting energy or macronutrient contributions to energy. Although 88% of observational trials reported energy intake, ≤ 47% reported macronutrient contributions to energy (Table 6). Despite the objective of many studies being to investigate cardiovascular-related events and biomarkers (Figure 5), only 5/28 and 7/28 interventions reported cholesterol or fiber intake, respectively. Vitamins, minerals, and carotenoid consumption were similarly reported infrequently, however observational reports described vitamin and mineral intake more frequently than interventions (Table 6).

**Table 6:** Studies reporting nutrient intake.^1^

Characteristic^1^	Count of primary reports, n (%)	
	Interventions 20 (100)	Observations 17 (100)
Energy	13 (65)	15 (88)
Fat, % E	15 (75)	8 (47)
Saturated fatty acids	8 (40)	6 (35)
MUFA	9 (45)	7 (41)
PUFA	8 (40)	7 (41)
Carbohydrate, % E	15 (75)	7 (41)
Dietary fiber	7 (35)	8 (47)
Total sugars	1 (5)	0
Protein, % E	15 (75)	6 (35)
Vitamins A, B, C, D, E or K	3 (15)	5 (29)
Minerals		
Calcium	2 (10)	4 (24)
Copper, iron, phosphorus, or zinc	1 (5)	2 (12)
Magnesium	1 (5)	6 (35)
Potassium	2 (10)	6 (35)
Sodium	2 (10)	5 (29)
Lutein/zeaxanthin, lycopene, or carotene	2 (10)	1 (6)
Other		
Cholesterol	5 (25)	2 (12)
Alcohol	2 (10)	8 (47)

% E, % of total energy. ^1^Includes either planned or actual nutrient intakes of the entire diet (measured at any point in time, baseline or follow-up) and excludes eight primary reports of acute studies on avocado-containing meals (not the entire diet), where the background diet would not be expected to have been reported. Intakes were not counted if reports provided partial information such as the composition of experimental meals but not intake of all other nutrients. Unless specified otherwise, counts include any unit described (g/kg, g/d, % E, etc.).

### Outcomes

Among intervention trials, lipids, nutrient bioavailability, CVD risk factors (e.g., blood pressure/flow/structure/function or biomarkers, excluding anthropometrics and lipids), anthropometrics, and glycemia were the most common outcomes measured (Figure 5). Nutrient bioavailability was measured with the greatest heterogeneity (34 variables were measured across 11 studies) compared to anthropometrics or glycemia, where four variables were measured across 10 trials. Outcomes whose number of variables measured exceeded the number of primary reports indicate high heterogeneity and where greater replication would be valuable. Trials lasting longer than one month tended to have larger sample sizes than those of shorter duration. Fourteen of 28 primary interventions (50%) were conducted within one month or shorter, 11 conducted for 1–3 months (39%), and three for 3–6 months (11%).

Among observational trials, anthropometry, CVD incidence, and glycemia were the most commonly measured. Most measures were analyzed in a cross-sectional design (11/17 primary observational reports), whether that be a national survey or otherwise. CVD incidence was not measured among any cross-sectional studies and was the outcome analyzed in the greatest number of cohort studies (47, 49, 69). Follow-up lengths for all outcomes were usually 10+ years, with only one cohort study (69) reporting a shorter follow-up period. Outside of cross-sectional studies only 1–2 studies have been conducted on any given outcome, representing a general lack of longitudinal data across all topics.

## Discussion

The objective of this scoping review was to systematically identify and characterize the body of evidence related to consumption of avocado and health, specifically in studies where the effect of the exposure was isolatable (compared to multi-component exposures containing avocado). Overall, research was primarily focused on cardiometabolic outcomes, with emerging domains including that of antioxidation, gut health, skin health, cognition, and immunity. Most studies evaluated participants in adulthood, typically overweight, and fairly balanced between sexes.

At first glance, it may seem that the large number of studies conducted on cardiometabolic outcomes renders the topic sufficiently studied. However, take the adjacent measure anthropometry for example. With respect to policy guidance, the USDA NESR protocol commonly uses a study duration cutoff of ≥12 weeks (sometimes as long as 6 months) in their eligibility criteria for studies on body composition (21, 70). In this scoping review, 10 primary interventions evaluated anthropometry (e.g., body weight, BMI, or body fat), two of which were conducted for ≥12 weeks (the Habitual Diet and Avocado trial, and the Effects of Avocado Intake on the Nutritional Status of Families trial) (71, 72). Both measured BMI, and one measured body fat (72). From this perspective, the size of the body of evidence quickly shrinks. Conversely, of the 11 studies on nutrient bioavailability, two have been measured on an acute scale (66, 68). Thus, there is more to learn regarding avocado’s impact on nutrient bioavailability on an acute scale. In general, nearly every topic outside of cardiometabolic-related outcomes represents a novel gap in the research, regardless of study design.

Despite only a few exclusively investigating the impact of avocado intake in populations with dyslipidemia, most studies sampled populations with elevated blood lipid concentrations. In some cases this issue is not serious and simply related to heterogenous populations and the report of participants across a range of healthy and elevated levels. For example, Alvizouri-Muñoz et al. (6), report subjects with 2.29–2.68 mmol/L LDL (88–103 mg/dL), thus subjects were in both the “healthy” and “elevated” categories. In other cases, reporting has serious implications for future meta-analysis. For example, Scott et al. (9) and Colquhuon et al. (73) do not explicitly report their subjects as having elevated lipids, yet subjects in both studies do. Unfortunately, this can create confusion, as a later meta-analysis (13) considered the participants in Scott et al. (9) as normocholesterolemic, despite meeting criteria for hypercholesterolemia based on LDL. For other studies lipid status was not discernible (74) given only the change from baseline, and not the actual baseline values, were reported. To avoid confusion, we recommend that (1) researchers avoid explicit uses of the term “healthy” unless well-defined, (2) researchers avoid publishing change-from-baseline-data without also reporting the raw baseline values, and (3) that future reviewers use the summary or baseline data to make such conclusions, rather than the terminology used by original authors.

### Translating between interventions and observational data

Observational studies typically measured a distinct population compared with interventions (either younger or older than interventions, with a typical or overweight but not obese BMI) and reported much lower intakes of avocado than interventions (Figure 4). Figure 5 indicates both the heterogeneity in outcomes measured between the two designs as well as the general dearth of observational studies. Ultimately, the observational data and interventions were conducted on separate populations, exposure levels, and endpoints, and may be ill-suited for substantiating the other’s findings. This gap represents a promising opportunity for future epidemiological studies. We recommend a review to identify existing observational data that measured avocado intake for further secondary analysis. Doing so would bolster the available epidemiological evidence across a wide range of exposures, populations, and endpoints, thus filling some gaps between intervention and observational trials. This may be achievable by specifically identifying observational data in Europe. Despite no published research on the topic from participants in Europe, in 2023 Europe accounted for the second highest global import of avocado (0.76 billion kg), 7x higher than the next largest import region (Canada at 0.11 billion kg), and 60% that of the U.S. (1.26 billion kg) (75). Thus, the high import of avocado into Europe makes the region a novel research population and potential source for secondary data on avocado consumption and health. Replicating observational studies conducted in the US in other countries in subjects of diverse race/ethnicity, socioeconomic position, gender identity, and health disparities would also satisfy methodological recommendations to help evaluate diet-health relationships stated in the Scientific Report of the 2025 Dietary Guidelines Advisory Committee (76).

### Availability of data for causal inference

The ability to make causal inferences about the impact of avocado intake and health is limited by a few characteristics of the body of literature, most notably the ability to account for dose–response effects and dietary compensation from avocado consumption.

First, one of 28 interventions (77) study evaluated a dose–response effect in a controlled trial. While doing so was more common among observational trials, intervention studies assessed high intakes of avocado (typically >50 g/d), whereas observational studies reported low intakes of avocado (typically 0–40 g/d), an underestimate that is likely related to only 2/17 observational primary reports calculating intake from all food sources of avocado (62, 63) (Figure 4). The difference in avocado consumption between interventions and observational trials complicates the comparison of findings from one study design to the other.

More importantly, there is limited understanding of dietary compensation from avocado intake. A third of trials provided avocado in a free-feeding context, and numerous trials either did not measure, analyze, or report nutrient intakes (Tables 5, 6). Such characteristics limit the ability to experimentally isolate or statistically evaluate the effect of the background diet. To overcome such limitations, we recommend the following. First, future interventions should consider using a dose–response design to identify if increasing avocado intake has a causal impact on health. Second, estimation of exposure to avocado in observational studies should estimate intake from all possible food sources (e.g., raw avocado pulp, guacamole, sushi, etc.). Estimation of avocado intake from food frequency questionnaires may be more accurate than from 24-h recalls or 3-7d food diaries in populations that consume avocado infrequently. Third, all studies (including those of acute nature) should measure and report energy, macronutrient, and micronutrient intake at the start and end of the trial or follow-up period. Doing so would enable quantification of potential dietary compensation and the ability to control for the background dietary pattern. Lastly, alongside macronutrient and MUFA intake, fiber should be considered a critical nutrient to control for. Fiber has a known, beneficial impact on cardiovascular health, and in the United States there are approved health claims related to fiber intake and coronary heart disease (21 CFR 101.77, 21 CFR 101.81). Yet, existing study designs of interventions were primarily designed to control energy, MUFA, and/or macronutrient intake (Table 4). Fiber intake could be used among interventions with a free-feeding design or observational trials as an additional measure to statistically control for.

## Strengths and limitations of the review

This review addresses many of the limitations present in previous publications, most importantly by focusing the review to studies wherein effects of intake could be isolated to quantifiable intake of avocado. Although omitting studies with multi-food interventions reduced the amount of in-scope literature, it enabled the identification of research from which causal relationships between avocado intake and health could be inferred. Although we did not systematically perform an umbrella review, we identified 36 articles (2, 3, 10, 43, 45–57, 62, 63, 65–67, 69, 77–89) that were not described in recent systematic reviews (12–16, 28, 29), indicating this review comprehensively captured the body of literature and is distinct from other systematic reviews. Second, the translation of studies in Spanish allowed inclusion of several studies (59, 60, 67, 77, 87), which is particularly critical given the cultural importance of avocado intake in Latin American populations. The use of backward citation screening identified two observational studies that were not identified from database searching (49, 84), both of which used terms related to fruit and vegetable intake but not related to avocado or MUFA in the abstract. This finding highlights the possibility that not all possible observational studies were identified, specifically, those articles that only report avocado intake in full-texts or supplementary data. Such articles will likely not be found by database searching unless using a very broad search strategy including fruit and vegetable terms and full-text review of epidemiological research with secondary outcomes on avocado intake, requiring a substantially larger screening process.

## Conclusions and future directions

We identified 58 articles comprising 45 unique studies on avocado intake and health. Research has been well-replicated among participants living in the Americas, in adults with high blood lipid concentrations, and on anthropometric and cardiovascular outcomes. Future studies to overcome the limiting factors of the research include: greater study of participants outside of the Americas across a wider age range, designs of interventions or epidemiological analyses that account for dietary compensation or other dietary patterns, and evaluation of the dose–response pattern. Such studies would help build causal evidence delineating whether avocado or the broader dietary patterns are responsible for the effects observed. Specific gaps in the research are listed below.

- There has been no research conducted in European or Asian regions (except Indonesia), with most research conducted in the U.S. and Mexico, typically related to cardiovascular outcomes.
- Adolescent or younger and senior populations have been understudied, especially with respect to interventions.
- Most studies sampled populations with elevated blood lipid concentrations (likely endemic to the general population), with a minority exclusively investigating the impact of avocado intake in dyslipidemic populations.
- There is a translational gap between observational and intervention studies according to the analyzed population’s age, weight, amount of avocado consumed, and endpoints measured. Further, some observational studies did not account for all sources of avocado intake.
- There is limited understanding of dietary compensation from avocado intake. Although several trials provided avocado in a free-feeding context, and numerous trials either did not measure, analyze, or report nutrient intakes.
- There were almost no dose–response evaluations in controlled trials.
- Although the number of interventions appears high, over half were conducted in short interventions of one month or less. Relatively little is known about the effects of avocado intake for longer than one month.

The full results are detailed in a publicly available interactive evidence map. The resource should help educate the broader community on the available research and enable more efficient and informed methods of planning future research.

## Data Availability

The original contributions presented in the study are included in the article/Supplementary material, further inquiries can be directed to the corresponding author. Protocols, Supplementary material, and raw data are freely available at https://github.com/Traverse-Science/Avocado-Evidence-Map.

## Footnotes

^1^https://scite.ai/home, last accessed 05/09/2024.

^2^https://sr-accelerator.com/#/spidercite, last accessed 04/02/2024.

^3^ https://www.covidence.org/

^4^Available at: www.covidence.org.
