# Supplementary material for: Exploring avocado consumption and health: a scoping review and evidence map
Source: Front Nutr. 2025 Feb 10;12:1488907. doi: 10.3389/fnut.2025.1488907 (PMC11847682; doi:10.3389/fnut.2025.1488907)
Supplement: Supplementary file 3 [file Supplementary_file_1.docx]

**Preferred Reporting Items for Systematic reviews and Meta-Analyses extension for Scoping Reviews (PRISMA-ScR) Checklist**

| **SECTION** | **ITEM** | **PRISMA-ScR CHECKLIST ITEM** | **Where reported** |
| --- | --- | --- | --- |
| **TITLE** | | | |
| Title | 1 | Identify the report as a scoping review. | In title |
| **ABSTRACT** | | | |
| Structured summary | 2 | Provide a structured summary that includes (as applicable): background, objectives, eligibility criteria, sources of evidence, charting methods, results, and conclusions that relate to the review questions and objectives. | In abstract |
| **INTRODUCTION** | | | |
| Rationale | 3 | Describe the rationale for the review in the context of what is already known. Explain why the review questions/objectives lend themselves to a scoping review approach. | Introduction paragraphs 3-4 |
| Objectives | 4 | Provide an explicit statement of the questions and objectives being addressed with reference to their key elements (e.g., population or participants, concepts, and context) or other relevant key elements used to conceptualize the review questions and/or objectives. | Introduction paragraph 4 |
| **METHODS** | | | |
| Protocol and registration | 5 | Indicate whether a review protocol exists; state if and where it can be accessed (e.g., a Web address); and if available, provide registration information, including the registration number. | Methods first paragraph |
| Eligibility criteria | 6 | Specify characteristics of the sources of evidence used as eligibility criteria (e.g., years considered, language, and publication status), and provide a rationale. | Heading “Eligibility criteria” and Table 1 |
| Information sources* | 7 | Describe all information sources in the search (e.g., databases with dates of coverage and contact with authors to identify additional sources), as well as the date the most recent search was executed. | Heading “Search strategy” |
| Search | 8 | Present the full electronic search strategy for at least 1 database, including any limits used, such that it could be repeated. | Supplemental Material |
| Selection of sources of evidence† | 9 | State the process for selecting sources of evidence (i.e., screening and eligibility) included in the scoping review. | Heading “Study screening, selection, and data extraction" |
| Data charting process‡ | 10 | Describe the methods of charting data from the included sources of evidence (e.g., calibrated forms or forms that have been tested by the team before their use, and whether data charting was done independently or in duplicate) and any processes for obtaining and confirming data from investigators. | Heading “Study screening, selection, and data extraction", Supplemental material |
| Data items | 11 | List and define all variables for which data were sought and any assumptions and simplifications made. | Heading “Study screening, selection, and data extraction", Supplemental material |
| Critical appraisal of individual sources of evidence§ | 12 | If done, provide a rationale for conducting a critical appraisal of included sources of evidence; describe the methods used and how this information was used in any data synthesis (if appropriate). | Not conducted |
| Synthesis of results | 13 | Describe the methods of handling and summarizing the data that were charted. | Heading “Data analysis and visualization”, Supplemental Material |
| **RESULTS** | | | |
| Selection of sources of evidence | 14 | Give numbers of sources of evidence screened, assessed for eligibility, and included in the review, with reasons for exclusions at each stage, ideally using a flow diagram. | Results first paragraph |
| Characteristics of sources of evidence | 15 | For each source of evidence, present characteristics for which data were charted and provide the citations. | All figures/Tables |
| Critical appraisal within sources of evidence | 16 | If done, present data on critical appraisal of included sources of evidence (see item 12). | Not conducted |
| Results of individual sources of evidence | 17 | For each included source of evidence, present the relevant data that were charted that relate to the review questions and objectives. | Supplemental Material and data |
| Synthesis of results | 18 | Summarize and/or present the charting results as they relate to the review questions and objectives. | All Figures/Tables |
| **DISCUSSION** | | | |
| Summary of evidence | 19 | Summarize the main results (including an overview of concepts, themes, and types of evidence available), link to the review questions and objectives, and consider the relevance to key groups. | Entire discussion section |
| Limitations | 20 | Discuss the limitations of the scoping review process. | Heading “Strengths and limitations of the review” |
| Conclusions | 21 | Provide a general interpretation of the results with respect to the review questions and objectives, as well as potential implications and/or next steps. | Heading “Conclusions and future directions" |
| **FUNDING** | | | |
| Funding | 22 | Describe sources of funding for the included sources of evidence, as well as sources of funding for the scoping review. Describe the role of the funders of the scoping review. | For review: Heading “Funding”  For sources of evidence: Figure 2B and supplemental data |

JBI = Joanna Briggs Institute; PRISMA-ScR = Preferred Reporting Items for Systematic reviews and Meta-Analyses extension for Scoping Reviews.

* Where *sources of evidence* (see second footnote) are compiled from, such as bibliographic databases, social media platforms, and Web sites.

† A more inclusive/heterogeneous term used to account for the different types of evidence or data sources (e.g., quantitative and/or qualitative research, expert opinion, and policy documents) that may be eligible in a scoping review as opposed to only studies. This is not to be confused with *information sources* (see first footnote).

‡ The frameworks by Arksey and O’Malley (6) and Levac and colleagues (7) and the JBI guidance (4, 5) refer to the process of data extraction in a scoping review as data charting*.*

§ The process of systematically examining research evidence to assess its validity, results, and relevance before using it to inform a decision. This term is used for items 12 and 19 instead of "risk of bias" (which is more applicable to systematic reviews of interventions) to include and acknowledge the various sources of evidence that may be used in a scoping review (e.g., quantitative and/or qualitative research, expert opinion, and policy document).

*From:* Tricco AC, Lillie E, Zarin W, O'Brien KK, Colquhoun H, Levac D, et al. PRISMA Extension for Scoping Reviews (PRISMAScR): Checklist and Explanation. Ann Intern Med. 2018;169:467–473. [doi: 10.7326/M18-0850](http://annals.org/aim/fullarticle/2700389/prisma-extension-scoping-reviews-prisma-scr-checklist-explanation).

# Readme

The following supplementary material describe the search strategy and data extraction protocols. The data have been made publicly available in two places.

1. GitHub and Zenodo
   1. Archived data: <https://doi.org/10.5281/zenodo.12824840>
      1. This provides a persistent link to the same data presented in the GitHub repository
   2. GithHub repository: <https://github.com/Traverse-Science/Avocado-Evidence-Map>
2. Tableau public
   1. <https://public.tableau.com/views/AvocadoEvidenceMapPublicStory/SEMPublicStory?:language=en-US&:sid=&:display_count=n&:origin=viz_share_link>.
   2. We have chosen not to allow download of the tableau workbook or underlying data. Please use the archived data instead, which is organized in a more intuitive format for those wishing to re-analyse the data or take a deeper look.

# Methods

## Search Strategy

### Searches October 17^th^, 2023

#### PubMed*

((avocado OR avo OR guacamole OR "Fuerte" OR "Hass") OR (("monounsaturated fat*" OR "monounsaturated fatty acids" OR fatty acids, monounsaturated[mh] OR MUFA) AND diet*) NOT review[pt])

Filters: Human only

Notes: Pubmed translations of avocado include the term “persea”.

#### Web of Science

(((avocado OR avo OR Persea OR guacamole OR Fuerte OR Hass ) AND human))

#### Scopus

TITLE-ABS-KEY ( "avocado" ) OR TITLE-ABS-KEY ( "guacamole" ) OR TITLE-ABS-KEY ( "Fuerte" ) OR TITLE-ABS-KEY ( "Hass" ) OR INDEXTERMS ( "avocado" ) AND ( LIMIT-TO ( DOCTYPE, "ar" ) ) AND ( LIMIT-TO ( EXACTKEYWORD, "Humans" ) )

#### CENTRAL

1 MeSH descriptor: [Persea] explode all trees

2 (avocado OR guacamole OR "Fuerte" OR "Hass")

#1 OR #2

### Searches After October 17^th^, 2024

Justification: Changes made to the search strategy reflect the following observations:

1. There were no in-scope studies that used the term “avo” but did NOT use the term “avocado”.
   1. The term “avo” was removed from all searches.
2. There were no in-scope studies on monounsaturated fatty acids (MUFA) that did not also use the term “avocado”
   1. Any terms related to MUFA were removed.
3. Only two studies were identified from backward citation screening or manual adds (1,2) and neither of them used terms related to avocado or MUFAs.
4. For pubmed: The scope was widened to remove the filter for publication type
5. The term “aguacate” was added to identify Spanish translations

Pubmed: For date restricted searches, the [Date - Create] field will be used, rather than the publication date, because this is the first date recorded in pubmed.

Ex: ("2023/10/01"[Date - Create] : "3000"[Date - Create])

#### PubMed*

(avocado OR guacamole OR "Fuerte" OR "Hass" OR "aguacate") AND ("2023/10/01"[Date - Create] : "3000"[Date - Create])

Filter: humans only

Notes: Pubmed translations of avocado include the term “persea”.

#### Web of Science

(avocado OR Persea OR guacamole OR Fuerte OR Hass OR aguacate) AND human

Filters: Human only

Set Dates with UI: index dates from 2023-10-17 to 2024-04-29

#### Scopus

TITLE-ABS-KEY ( ( "avocado" ) OR ( "aguacate" ) OR ( "guacamole" ) OR ( "Persea" ) OR ( "Fuerte" ) OR ( "Hass" ) ) OR INDEXTERMS ( "avocado" ) AND PUBYEAR > 2022 AND PUBYEAR < 2025 AND ( LIMIT-TO ( DOCTYPE, "ar" ) ) AND ( LIMIT-TO ( EXACTKEYWORD, "Human" ) )

Can’t set a date for a specific day, the year will be used.

#### CENTRAL (trials only)

1 MeSH descriptor: [Persea] explode all trees

2 (avocado OR guacamole OR "Fuerte" OR "Hass" OR “aguacate”)

#1 OR #2

The UI will be used to set dates

## Data Extraction Protocols

### Review sheet

This sheet represents variables collected on a “one study per-row” basis.

| **Supplemental Table 1.** Study-level variables | | | |
| --- | --- | --- | --- |
| **Variable** | **Notes** | **Type** | ***Example(s)*** |
| Index | Internal reference number | Numeric | 1,2,3 |
| PMID | The PubMed Identifier | Free text | 31616932 |
| DOI | The digital object identifier for the paper | Free text | 10.1371/journal.pone.017264 |
| Citation | Last name of first author and year published | Free text | Wang 2020 |
| Parent Study | Unique identifier for multiple articles related to the same study.  Clinical trial registry number, cohort name, or other.  If no Trial # or cohort name is available, the citation is used. | Free text | Persea americana for Total Health (PATH) Study; NCT01271829; Khor 2014 |
| Main | For multiple citations related to the same parent study, this designates:  Main: The “main” or “primary report”, typically the first publication related to the parent study.  Unique: If the population or analysis is different from the “Main” study.  Not-unique: This article may conduct the same analysis on the same population as the main study, but on different outcomes. | Categorical | Main;Unique;Not unique;NA |
| Authors | Full list of authors | Free text | Wang L, Tao L, Hao L, Stanley TH, Huang KH, Lambert JD, Kris-Etherton PM |
| Year | Year of publication | Date | 2006 |
| Journal | Name of journal where paper was published - full title, not abbreviated | Free text | American Journal of Clinical Nutrition |
| Title | Full title of the article | Free text | A moderate-fat diet with one avocado per day increases plasma antioxidants and decreases the oxidation of small, dense LDL in adults with overweight and obesity: A randomized controlled trial |
| Abstract | Full abstract of the article | Free text | - |
| Population Region | The country where subjects were studied, not the country of origin of the authors. | Multi-categorical, Qualitative | USA; Mexico; Australia; South Africa |
| Age Bin | Age categories of all study participants. Categories are defined below. Categorizations are based off of the mean age reported. This is based on values from the group-level datasheet.  Infant (<3 years) (used as a shorthand, even though toddlers are not infants) Child (3-11 years) Adolescent (12-17 years) Adult (18-64 years) Senior (65+ years) | Multi-Categorical, Qualitative | Adolescent or younger; Adult; Senior |
| Target population | Description of health status of participants at baseline; If subjects were not recruited based upon specific disease-related criteria, they were listed as “Healthy” or “None” or “General population”. Overweight and obesity are not considered diseases for this variable, these are captured by a separate variable. Old age should be captured in the Age category, not here. Sex should be captured in subject characteristics.  In later cleaning steps similar health statuses may be grouped together for ease of tabulation/visualization. | Free-text | Elevated LDL-C; Diabetic; hypercholesterolemic; Hookworm; insulin resistant. |
| BMI Bin | Description of weight/BMI of participants at enrolment, based on the mean BMI reported of groups or sub-groups at any time during the study. This is empty if BMI is not provided. This is based on values from the group-level datasheet.  BMI (adults)  18 ≤ Typical < 25  25 ≤ OW < 30  <30 OB | Multi-categorical, Qualitative | Underweight;Healthy weight, Overweight or obese |
| Cholesterol bin | A binning variable detailing whether subjects had healthy or elevated levels of cholesterol based on their HDL, LDL, triglycerides, or total cholesterol. This is based on values from the group-level datasheet. Classified according to any of HDL, LDL, or total cholesterol, where healthy was designated (all in mg/dL) as 125-200 total cholesterol, < 100 LDL cholesterol, > 50 HDL cholesterol, and < 150 triacylglycerols, using ranges set at https://medlineplus.gov/cholesterollevelswhatyouneedtoknow.html. Although HDL > 40 is considered healthy for males, most studies did not separate the data by sex. Healthy concentrations were based on guidelines for women given greater representation of females across studies. | Multi-categorical, Qualitative | Healthy;Elevated |
| %F bin | A binning variable detailing the proportion of the total sample size that was male or female. This is based on the count of males/females listed in the group-level datasheet.  Mostly female (> 60% female)  Balanced (40-60% female)  Mostly male (< 40% female) | Categorical, qualitative | Mostly female; balanced |
| SS, Assigned | Parallel-arm: Total sample size across all arms at randomization/enrollment.  Cross-over: Total subjects enrolled, regardless of the number of arms.  Cohort: Total subjects enrolled into cohort  Survey and cross-sectional: NA | Numeric | 45 |
| SS, Completed | Number of subjects that completed a trial/cohort or that were analyzed. Take the highest number, even though multiple outcomes/analyses may have different sample sizes, or there was further dropout between an intent-to-treat or per protocol analysis.  Parallel arm: Total sample size across all arms at analysis/completion.  Cross-over: Total subjects at analysis/completion, regardless of the number of arms.  Cohort: Total subjects at analysis/completion.  Survey and cross-sectional: Total subjects analyzed across all arms/sub-groups. | Numeric | 40 |
| Study Type | Overarching study type, whether observational or intervention. | Categorical, Qualitative | Intervention; Observation |
| Study Design | The general design of the study. | Categorical, Qualitative | Intervention: Crossover, parallel-arm, single-arm, or other.  Observation: Survey, cohort, case-control, or other |
| Study Type Notes | An optional column for any notes related to the study design | Free text | Randomized;investigator-blinded;parallel-arm;controlled trial |
| Max Intervention Length, days | For intervention trials only. The length of time subjects were exposed to the treatment, not the length of the study itself. This number should exclude run-in and washout periods. It only represents the length of a single arm in a cross-over, not multiple arms.  For acute studies, this is 1 day. | Numeric | 35, 84 |
| Intervention Length | A categorized version of the previous variable | Categorical | Acute; 2-30d, 1-3 mo |
| Max Observational Follow-up Length, years | The max length of time subjects were followed up with. For cross-sectional studies, this is NA | Numeric | 180; 365 |
| Observational Follow-up | A categorized version of the previous variable | Categorical | Cross-sectional;7-15 years |
| Form When Consumed | The method that avocado was prepared/provided for consumption.  For observational studies, this is all food sources of avocado. | Free text | Fresh, chunks; Guacamole; Fresh pulp; Not specified |
| Cultivar | The cultivar of avocado consumed  *As of 4/2/2024 these data are not verified.* | Free text | Hass; Sharwill; Florida |
| Intended Energy Balance of Subjects | Did the study intend for subjects to consume below, at, or above their energy requirements? If the subjects were allowed to consume an *ad libitum* amount of energy, or were allowed to consume their habitual diet, this is “no intent”. This should be entered for the period of exposure to the treatment, ignoring any previous run-in, standardization, or weight-loss periods prior to the intervention or during wash-out periods. | Multi-categorical, Qualitative | Weight loss; Weight Maintenance; Weight gain; No intent |
| Intended energy balance between groups | Were the diets designed between groups to be isocaloric?  If there was no report of intent to match energy intake between groups, enter “no intent”.  In cases where all groups are provided energy on a BW basis, it is assumed that energy intake is intended to be equal between groups, unless the groups are intentionally of different weights. | Multi-categorical, Qualitative | Hypo; isocaloric; hypercaloric; No intent |
| Constant Macros, detailed | Whether or not the % of energy from protein, fats, and carbs was consistent between groups or not. Options are Yes/No/NA?Not reported, but additional text is allowed to indicate if the macros were constant on purpose or not. | Free text | Yes (unintentionally) |
| Constant Macros | A cleaned and binned version of Constant Macros, detailed variable | Multi-categorical, Qualitative | Y;N;Not specified;NA |
| Meal control, detailed | Whether or not subjects were allowed to consume the treatment/exposure *ad libitum* or according to a controlled feeding design.  Controlled: Provision of or prescribed diets/menus for all food/meals, or of meals in acute studies.   Partial ad lib: Subjects are allowed to consume what they want as long as they hit macronutrient or other related targets (energy intake). Provision of some meals, with ad libitum consumption of other meals.  Ad libitum: Subjects consume their habitual diet, are free-living/consuming, or specified to eat ad libitum. This is true even if instructed to consume 1 avocado/day.  Provision of dietary guidance was not considered a restriction (controlled or partial ad lib), unless the guidance was to ensure subjects adhered to nutrient targets. | Multi-categorical, Qualitative | *Ad libitum*; Controlled (6-d menu); Partially controlled |
| Meal control | A binned version of the previous variable | Multi-categorical, Qualitative | *Ad libitum*; Controlled; Partially controlled |
| Comparators, detailed | A free text description of the available comparators | Free text | High carbohydrate diet (no avocado or olive oil);  Low fat diet, 6-7% energy from grain, or moderate fat diet containing oleic acid. |
| Comparator clean | A shorthand description of the comparators | Free text | Corn oil;No avocado;Dose-response |
| Comparator model | Whether avocado was supplied with replacement of other foods/nutrients, or top-of the usual diet. | Multi-categorical, Qualitative | Replacement; Addition |
| Measurement Methods for intake | A free text description of what methods were used to measure nutrient/food intake | Free text | 6-day rotating menu provided, nutrient content of the formulated diets analyzed using food processor SQL software, subjects weighed to assess diet compliance |
| Nutrient ascertainment, detailed | A categorical description of how nutrient intake was quantified. Do not need details for what software were used or how compliance/adherence was handled. | Categorical, qualitative | Prescribed; Prescribed, not measured; 24-hr recall; FFQ; 7-d diet record |
| Nutrient ascertainment, binned | Further categorization of the nutrient ascertainment variable. | Categorical, qualitative | Prescribed; 24-hr recall; FFQ; Other |
| Funding Organization, reported | Description of who funded the research or other financial disclosures. | Free text | National Heart, Lung, and Blood Institute;Avocado Nutrition Center |
| Funding Organization, cleaned | A cleaned version of the funding organization, combining synonymous organizations or using organization abbreviations. | Multi-categorical, Qualitative | NHLBI;Hass Avocado Board |
| Funding Organization, binned | A further categorized version of the funding organization into: commodity boards (hass avocado board), research institutes (e.g., USDA, NIH), universities, and other (companies, hospitals, etc.) | Multi-categorical, Qualitative | Research institute; Commodity board |

### Data Extraction

Data collected at the outcome level, annotated on a per outcome, per group, per timepoint, per analysis conducted basis.

| **Supplemental Table 2.** Outcome level variables | | | |
| --- | --- | --- | --- |
| **Dosing, outcomes, dietary, and subject characteristics** | | | |
| **Variable** | **Notes** | **Type** | ***Example(s)*** |
| Index | Internal reference number. | Numeric | 1,2,3 |
| Citation | Last name of first author and year published. | Free text | Wang 2020 |
| Study Type | Overarching study type, whether observational or intervention. | Categorical, Qualitative | Intervention; Observation |
| Primary Report | “labelled as “parent study/clinical trial”” in Tableau viz. |  |  |
| Source | The place in the manuscript this variable was taken from. If it was analysed and not found in the manuscript, enter “estimated”. | Categorical | Methods; Results; Fig X; Table X; Supplemental Text; Supplemental Table/Figure X; Estimated |
| Group ID | The name/label of the group that data are extracted from. | Free text | Avocado inclusive; Control; Treatment; All |
| Sub-group 1 | Whether the data are a sub-group of the larger population. Studies may have all of the groups within a treatment stratified by age (senior vs adult vs child), weight (obese vs overweight), cardiovascular risk (high vs low), or some other factors.  Sub-group should *not* be used to describe the analytical population, if different models/adjustments were made, or if an analysis was a between/within-group analysis. | Free text or Categorical | Children; overweight subjects; subjects at risk for disease; |
| Analytical Population | **Avocado intake:**  Whether or not intake represents the intended intake of avocado (planned), or the amount adjusted for actual intake (actual). Actual intakes will usually be represented by an average + error.  **Intervention outcomes, dietary intake, and subject characteristics:**  Whether the data pertain to an intent-to-treat (ITT), per protocol (PP), as-treated, or other population. [See here](https://www.ncbi.nlm.nih.gov/pmc/articles/PMC5654877/) for a description.  It may not be possible to accurately classify the type of analysis when the authors do not describe it in some terms. Use the below as a guide.  Examples  **ITT:** Subjects are analysed according to the group they are originally assigned. Once a subject is enrolled into a group, even if they don’t receive the treatment (death, lack of compliance, lack of adherence, etc.), they are still included in the analysis.  *Hint:* You can tell if the data are ITT because the number of subjects in all arms of the trial is the same as the number of subjects enrolled into each arm. It is possible that some subjects dropped out, but they would typically be “filled in” through processes called imputation, mixed models, repeated measures, or last observation carried forward.  **PP:** Only the subjects who actually received the intervention assigned are analysed. Other names include “efficacy”, “explanatory analysis”, or “analysis by treatment administered”. If a subject received the wrong intervention and was analysed accordingly, this would be an “as-treated” basis which is a slightly different analysis. For our purposes, we consider them the same as a PP.  *Hint:* The sample size of the arms (at analysis) is less than the # of subjects assigned to those arms. The analysis is adjusted by adherence (intake of avocado, or provided meals).  Example: Wang 2015 (index 8). The baseline (AAD) data is ITT, because data for all 45 subjects was analyzed. However, the outcomes were PP because there were 42-43 subjects in each arm.  Pacheco 2022 (Index 48) demonstrates the difference between ITT and PP. Their PP 1) had 31/35 subjects and 2) adjusted for adherence.  **Observational data**: No entry | Categorical | ITT; PP; Not specified; Planned;Actual |
| Comparison | **Intervention**  This variable should be used to denote whether a 1) between-group, 2) within-group, 3) between-group difference of the within-group change was made or 4) the variable was not a comparison.  “Change in” or “change from baseline” represents a within-group comparison.  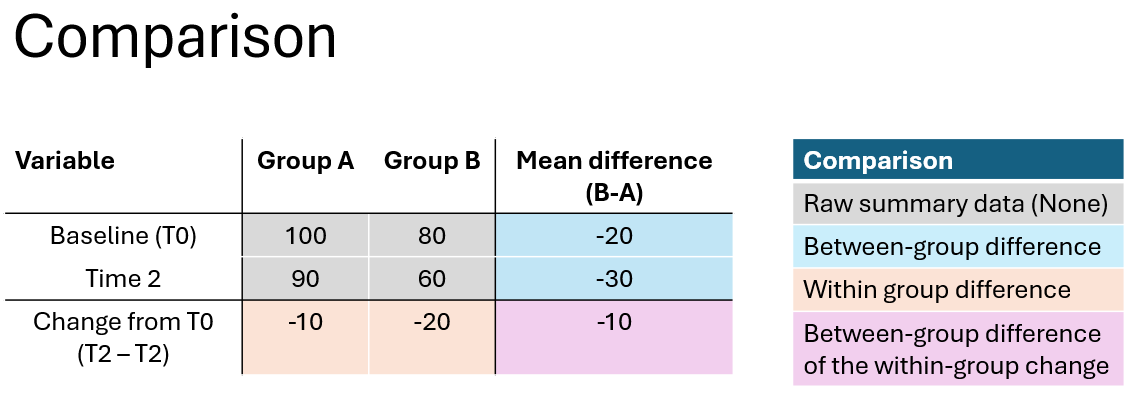  **Observational:** This is the “reference group” that a value is compared to (for outcomes like relative risk, odds ratios, hazard ratios, etc.). It may appear similar to the table above. If there is no reference group, the entry is likely “summary data”. | Categorical | Between; within; between-within;None |
| Measure Domain | A top-level domain that clarifies if the data pertain to dietary intake, subject characteristics, or outcome data. | Categorical | *Dietary; Subchar; Outcomes* |
| Measure Subdomain | Broad outcome domain for each outcome measure. This will be assigned by the review manager. | Categorical | *Lipids; Immune; Macronutrient; Micronutrient; Age; Anthropometry* |
| Measure,  Binned | A binned version that groups measures reported according to different sub-outcomes or measurement points into a single variable. | Free text | Triglycerides II and IIa = Triglycerides  Carotene at 9 and 12 hours = Carotene  Convenience of diet (less, more) = Convenience of diet  *Large, medium, small VLDL = VLDL* |
| Measure, Cleaned | A cleaned version of the reported measure, such that differences in formatting/spelling and synonymous terms are cleaned and grouped.  Subgroup versions of the same variable do NOT need to be cleaned. Thus insulin at 5 and 8 hr AUC will not be grouped into one variable.  These may be grouped or titled differently in figures/tables in Tableau for ease of reporting. | Free text | Women and female = female  a-TNF and α-TNF = α-TNF |
| Measure, Reported | The name of the outcome measured, as reported by the authors.  For outcomes in acute studies where samples were measured multiple times within a day (e.g., insulin over 5 hrs, or measured once but after 3-hr), enter the time under here, not in the time column.  Measures with a time-related unit less than one day are reported here. | *Free text* | *Insulin AUC at 5hrs; Triglycerides II; fat mass index* |
| Sample Type | Where was the outcome variable measured or sourced from, as reported in the article if possible.  If circulating insulin was measured, but plasma/serum wasn’t specified, enter “blood”.  Grouping will be performed in tableau, this can be specific. | Categorical | Saliva;Urine;Serum; Plasma;Blood;Feces |
| Time | The timepoint/visit at which data were collected from. If subject data was collected at baseline and 8 weeks later, this would be listed as “0” and “8”.  For outcomes in acute studies where samples were measured multiple times within a day (e.g., insulin over 5 hrs, or even if just measured once but after a few sec/min/hour), enter the time under “Measure, Reported”, not in the time column., and enter “acute” here.  For diet-related parameters that may not differ over time (such as the background diet), the phrase “background diet” may be entered. For baseline variables, the term “baseline” can be used.  **Observational data**: No entry, use the max follow-up time. | Numeric OR categorical | 3;6;10; Background diet; Subject characteristics; Acute |
| Time unit | Unit associated with the time.  There should be no entry of times less than one day here (no seconds, minutes, or hours).  **Observational data**: No entry | Categorical | Yr;mths;wks;d |
| Time, weeks | The same value as “Time” provided in weeks | Numeric OR categorical | 3;6;10 |
| n | Number of subjects this variable pertains to.  n applies to the subgroup if available, otherwise the group ID  -a mean taken on 45 subjects is n = 45  Subgroups  - If the outcome only relates to the count of a specific subgroup, use the total within that subgroup.  e.g., If there are 50 subjects in the group ID, but a variable is sub-grouped into 30 old and 20 young subjects, the n is 30 for the old subgroup. Then, if the variable reports that there are 10 old subjects at high and 20 old subjects at low risk of diabetes, the n is still 30, but the amount of subjects at risk go as a “Count” data under the Value and Statistic columns. | Numeric | 61 |
| Statistic | Was the value reported as a mean, median, absolute value, percentage, count, or other value. For count-related, use “count” as the amount and “Percentage” for % as it relates to the count.  If a study has mean AND median, only grab the mean.  For observational studies, beta coefficients will go on their own row, with beta as the “statistic” related to the value, instead of a mean/median. | Categorical | Mean; Median; Count; Frequency; |
| Unit | Unit of measure for each outcome measure | Categorical | mg/dL; g/kg; IU/L |
| Value | The value pertaining to the statistic | Numeric | 0.7; 100;245.5 |
| SE | Reported standard error *– convert to SD using the formula provided*. If SD is provided, enter “NA” here, which will be converted in data cleaning steps.  $SE=\frac{SD}{\sqrt{n}} SD=SE*\sqrt{n}$ | Numeric | 0.7; 100;245.5 |
| SD | Reported standard deviation | Numeric | 0.7; 100;245.5 |
| Lower | The lower limit of a provided range | Numeric | 25 |
| Upper | The upper limit of a provided range | Numeric | 327 |
| Range Type | Was the range an IQR, min-max, 95% CI, or some other specification? | Categorical | IQR; 95% CI, min-max; etc. |
| UNIT CONVERTED | A conversion of the “Unit” column into a new unit, such as converting mmol to mg. | Categorical | mg;mmol;kg;mL |
| SE CONVERTED | A conversion of the “SE” column based on the “UNIT CONVERTED”. | Numeric | 0.7; 100;245.5 |
| SD CONVERTED | A conversion of the “SD” column based on the “UNIT CONVERTED”. | Numeric | 0.7; 100;245.5 |
| Lower CONVERTED | A conversion of the “Lower” column based on the “UNIT CONVERTED”. | Numeric | 25 |
| Upper CONVERTED | A conversion of the “Upper” column based on the “UNIT CONVERTED”. | Numeric | 327 |
| Conversions | The fields Unit, Value, SE, SD, Lower, and Upper will be converted to either a standardized/common unit for ease of comparison. This variables lists manual notes made on conversions for select outcomes. This is not intended to be exhaustive. | Numeric | 0.7; 100;245.5 |

### Collection of subject characteristics data

Sometimes the subject characteristics are not presented in the same groupings as the outcomes. The below flowchart indicates what data to collect. This procedure limits the amount of data extraction necessary. Over-extraction will not hurt, but it is superfluous.


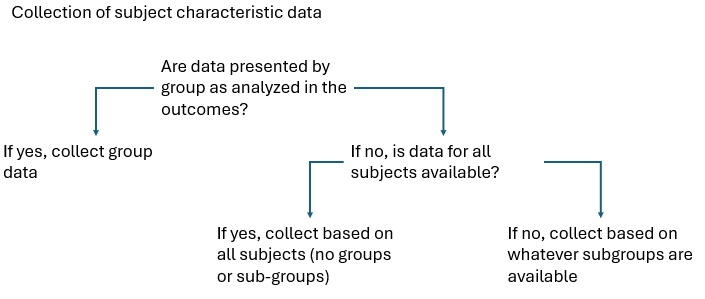


### Collection of time-related data

To avoid confusion, “time” will refer to the timepoints/visits/follow-ups reported in an article. It will be measured in units not smaller than days. For variables that are sampled over time within a day (on the scale of seconds to hours, or morning/evening), these will become a part of the “measure, reported” column. Baseline should be recorded as “0” and “weeks” in the “time” and “time unit” columns. Variables that have no associated time (e.g., subject characteristics, background diet) will be entered as “NA”.

For area under the curve (AUC) variables: Values for individual timepoints are NOT needed if AUC is available.


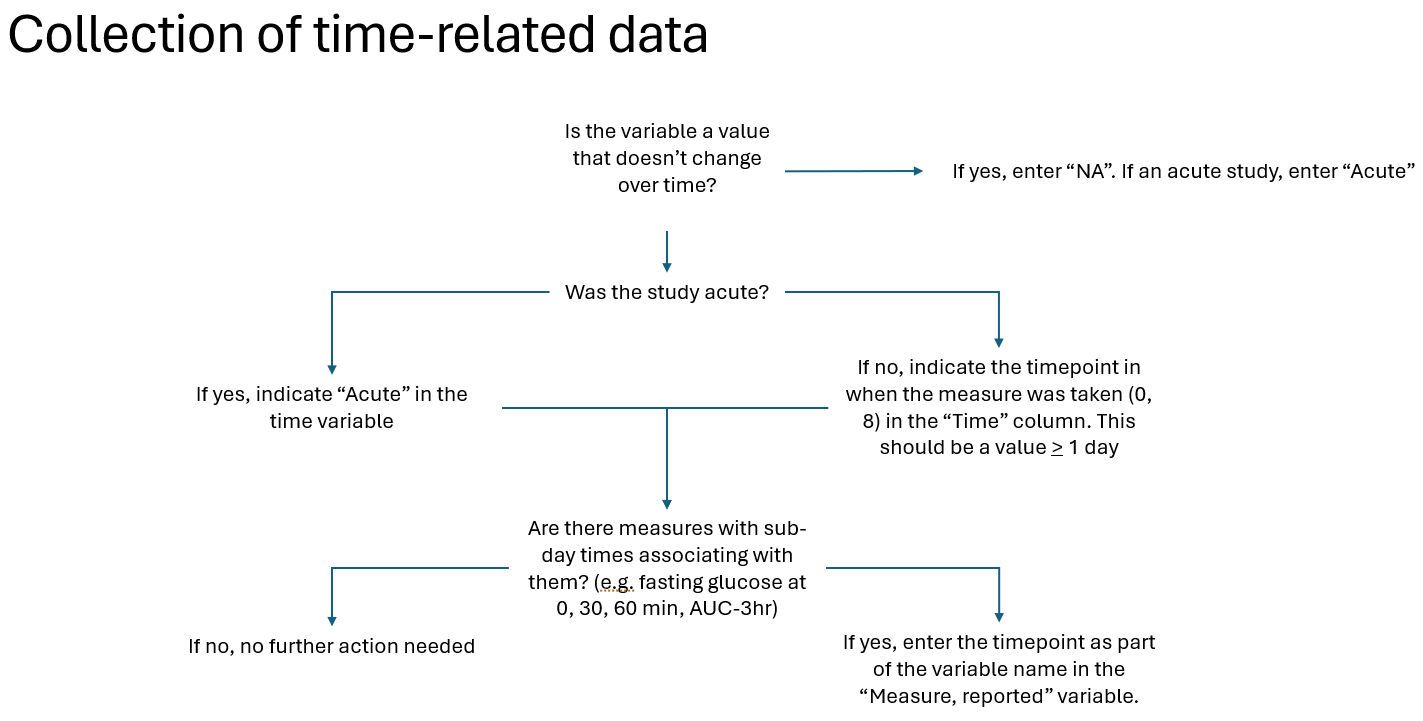


### Collection of dietary characteristics

The composition of individual meals, or the summation of all meals where provided, will not be extracted. Only nutrient intake of the full background diet (planned or actual intakes) will be extracted.

## Conversions

Time-related conversions: 1 month = 30 days, 1 week = 7 days

Mass/volume-related conversions: Standard transformations for mg-kg or ml-L will be used (multiply/divide by 1000).

Molar conversions: Molar masses will be used to convert between moles and mg.

Dietary energy conversions: 1 g of carbohydrate/protein = 4 kcal, 1 g of fat = 9 kcal. This is only necessary for conversions of avocado intake from a %E basis to g/d basis, precision at 0 significant figures is sufficient. will be used for conversions between g and kcal for protein (4), carbohydrate (4), and fat (9 kcal). Where possible, macronutrient intake will be converted to a %E basis.

Avocado intake: Where available, the average weight of the avocado (as described, be it pulp or whole) reported in the study will be used. When not available, one medium avocado is assumed to weigh 150g = 1 cup = 3 servings. This is consistent with FoodDataCentral ID: [171705](https://fdc.nal.usda.gov/fdc-app.html#/food-details/171705/nutrients) for cubed avocado, the [nutrition facts label](https://loveonetoday.com/nutrition/avocado-nutrition-facts-label/), and commonly reported in the literature (3–5). If only ranges are provided, the mean value of the bounds will be used (e.g., mean of 350 g for a range of 200-500 g).

Where a categorical range is provided (e.g., 1-2/week) that is not accompanied by a quantitative scale, the average between bounds will be taken. For limitless upper bounds, the lower bound will be used. For bounds starting at zero, zero will be chosen [for consistency with other reports such as (6)]. For example:

| **Supplemental Table 3.** Example quantification for categorical exposures | | | | |
| --- | --- | --- | --- | --- |
| **Range** | **Value** | **Lower** | **Upper** | **Unit** |
| Never or <=1/month | 0 | 0 | 1 | Servings/month |
| 2-3/month | 2.5 | 2 | 3 | Servings/month |
| 1/wk | 1 | 1 | 1 | Servings/week |
| 2-4/wk | 3 | 2 | 4 | Servings/week |
| 3+/wk | 3 | 3 | Undefined | Servings/week |
| 5+/wk | 5 | 5 | Undefined | Servings/week |

| **Supplemental Table 4.** Conversion of categorical intakes to quantitative values in observational trials | | | | |
| --- | --- | --- | --- | --- |
| **Citation (label in database)** | **Author Conversion** | **Notes** | **Our conversion** | **Energy intake?** |
| Borgi 2016 | 1 serving = ½ avocado | No g conversion listed.  Assume 1 cup = 150g cubed avocado (171705), ½ cup = 75 g | 1 serving = ½ avocado = 75 g | Available |
| Carris 2024 | Non-consumers  Low consumers (1-3 times/month)  High consumers (at least 1/wk) | No g conversion listed.  Assume similar g amount as Cheng 2023. | 1 serving = 1/3 avocado = 50 g | Not available |
| Cheng 2021 | g/d amount provided | NA | No conversion needed | Available |
| Cheng 2023 | pg 2108 last line:  1 serving = 1/3 medium avocado = 50 g    Pg 2106 Dietary assessment:  1 small avocado = 0.5 serving  1 medium avocado = 1 serving  1 large avocado = 1.5 servings | Description on pg 2106 appears inconsistent with pg 2108. | 1 serving = 1/3 avocado = 50 g | Available |
| Cheng 2024a | 1 serving = ½ avocado = ½ cup (Same as Ericsson) | No g conversion listed.  Assume 1 cup = 150g cubed avocado (171705), ½ cup = 75 g | 1 serving = ½ avocado = 75 g | Available |
| Cheng 2024b | g/d amount provided | NA | No conversion needed | Available |
| Ericsson 2022 | Pg 3, Assessment of avocado consumption:  1 serving = ½ cup avocado | No g conversion listed.  Assume 1 cup = 150g cubed avocado (171705), ½ cup = 75 g. | 1 serving = ½ avocado = 75 g | Available |
| Fulgoni 2013 | g/d amount provided | NA | No conversion needed | Available |
| Guan 2022 | g/d amount provided | NA | No conversion needed | Available |
| Heskey 2019 | g/d amount provided | NA | No conversion needed | Available |
| Jackson 2012 | g/d amount provided | NA | No conversion needed | Available |
| Mao 2019 | 1 serving = ¼ median | Assume median = medium avocado. | 1 serving = 1/4 avocado = 37.5 g | Available, but not on a basis that is related to the outcome. Have avocado and energy intake for quintiles of fruit and vegetables, but not energy for levels of avocado intake. |
| Monge 2023 | Abstract, pg 2 Dietary assessment  1 serving = half an avocado, defined by NHANES (Fulgoni 2013) | The Fulgoni article states 70 g is ~1/2 medium avocado, which we assume is 75 g | 1 serving = ½ avocado = 75 g | Available |
| Monroe 2007 | Avocado Quintiles, g/1000kcal | Weighted average across groups kcal = 1995 kcal/d | 1 avocado = 240 kcal and 150 g | Available |
| Morales 2023 | Units/week  0-.5, .5-3, >3 | No g/serving conversion listed.  It’s unlikely that the authors intend a unit to indicate a serving, as other foodgroups (vegetables, legumes, nuts, lean meat) are presented on either a serving/d or teaspoon/d basis. One serving would be ½ an avocado.  Assume 1 unit = 1 avocado = 150 g  Values convert to 5-64 g/d, which is not unusual, especially for a Latin American regions. | 1 unit = 1 avocado =150 g | Not available |
| O’Neil 2017 | g/d amount provided | NA | No conversion needed | Available |
| Pacheco 2022a | g/d amount provided | NA | No conversion needed | Available |
| Probst 2024 | g/d amount provided | NA | No conversion needed | Available |
| Segovia-Siapco 2021 | g/d amount provided | NA | No conversion needed | Available |
| Senn 2023 | 1 serving = ½ an avocado = 6.7 g MUFA  1 serving = 1/3 medium avocado = 50 g | 150 g (cubed) has 14.7g MUFA, which divided by 2 is 7.35 g MUFA | 1 serving = 1/3 avocado = 50 g | Not available |
| Wood 2023 | Table S1  1 small avocado/guac = 0.5 serving  1 medium avocado/guac = 1 serving  1 large avocado/guac = 1.5 servings | No g conversion listed.  Assume similar g amount as Cheng 2023. | 1 serving = 1/3 avocado = 50 g | Available |

**Conversions from an energy-basis to a gram/d basis**

Some studies may require conversion from a % energy/fat basis. For example, if subjects on a 2,000 calorie diet consumed 30% fat, with 75% of the fat from avocado, then the E % from fat as avocado is 22.5% (30%*0.75). This can be back calculated to g avocado/d with the following:

$$1. \frac{2000 kcal}{day}* \frac{30 kcal fat}{100 kcal}=*\frac{600 kcal fat}{d}$$

$$2. \frac{600 kcal fat}{d}*\frac{75 kcal avo fat}{100 kcal fat}=\frac{450kcal avo fat}{d}$$

$$3. \frac{450kcal avo fat}{d}*\frac{1g avo fat}{9kcal avo fat}*\frac{100g avo}{14.7 g avo fat}=\frac{\boldsymbol{340.14}\boldsymbol{g avo}}{\boldsymbol{d}}$$

$$Full: \frac{2000 kcal}{day}* \frac{30 kcal fat}{100 kcal}*\frac{75 kcal avo fat}{100 kcal fat}*\frac{1 g avo fat}{9 kcal avo fat}\frac{100 g avo}{14.7 g avo fat}=\frac{\boldsymbol{340.14 g avo}}{\boldsymbol{d}}$$

Shorthand: Energy*%E fat*%fat from avocado/9*100/14.7

# References

1. Borgi L, Muraki I, Satija A, Willett WC, Rimm EB, Forman JP. Fruit and Vegetable Consumption and the Incidence of Hypertension in Three Prospective Cohort Studies. Hypertension. 2016;67(2):288–93.

2. Mao X, Chen C, Xun P, Daviglus ML, Steffen LM, Jacobs DR, et al. Intake of Vegetables and Fruits Through Young Adulthood Is Associated with Better Cognitive Function in Midlife in the US General Population. J Nutr. 2019;149(8):1424–33.

3. Senn MK, Goodarzi MO, Ramesh G, Allison MA, Graff M, Young KL, et al. Associations between avocado intake and measures of glucose and insulin homeostasis in Hispanic individuals with and without type 2 diabetes: Results from the Hispanic Community Health Study/Study of Latinos (HCHS/SOL). Nutr, Metab Cardiovasc Dis. 2023;33(12):2428–39.

4. Fulgoni VL, Dreher M, Davenport AJ. Avocado consumption is associated with better diet quality and nutrient intake, and lower metabolic syndrome risk in US adults: results from the National Health and Nutrition Examination Survey (NHANES) 2001–2008. Nutr J. 2013;12(1):1.

5. Cheng FW, Ford NA, Wood AC, Tracy R. Avocado consumption and markers of inflammation: results from the Multi-Ethnic Study of Atherosclerosis (MESA). Eur J Nutr. 2023;62(5):2105–13.

6. Pacheco LS, Li Y, Rimm EB, Manson JE, Sun Q, Rexrode K, et al. Avocado Consumption and Risk of Cardiovascular Disease in US Adults. J Am Hear Assoc. 2022;11(7):e024014.
